# Risk knowledge of people with relapsing-remitting multiple sclerosis – Results of an international survey

**DOI:** 10.1371/journal.pone.0208004

**Published:** 2018-11-29

**Authors:** Andrea Giordano, Katrin Liethmann, Sascha Köpke, Jana Poettgen, Anne Christin Rahn, Jelena Drulovic, Yesim Beckmann, Jaume Sastre-Garriga, Ian Galea, Marco Heerings, Peter Joseph Jongen, Eik Vettorazzi, Alessandra Solari, Christoph Heesen, on behalf of the AutoMS group

**Affiliations:** 1 Service of Neuroepidemiology, Fondazione IRCCS Istituto Neurologico Carlo Besta, Milan, Italy; 2 Department of Psychology, University of Turin, Turin, Italy; 3 Institut für Neuroimmunologie und Multiple Sklerose, Universitätsklinikum Hamburg-Eppendorf, Hamburg, Germany; 4 Unit of Health Sciences and Education, Faculty of Mathematics, Informatics and Natural Sciences, University of Hamburg, Hamburg, Germany; 5 Pediatrics and Medical Psychology, University Medical Center Schleswig-Holstein, Campus Kiel, Kiel, Germany; 6 Institute for Social Medicine and Epidemiology, University of Lübeck, Lübeck, Germany; 7 Department of Neurology, University Medical Center Eppendorf, Hamburg, Germany; 8 Institute of Neurology, Clinical Center of Serbia, University of Belgrade, Belgrade, Serbia; 9 Department of Neurology, Faculty of Medicine, Ataturk Training and Research Hospital, Izmir, Turkey; 10 Multiple Sclerosis Centre of Catalonia (Cemcat), Neurology-Neuroimmunology Department, Vall d’Hebron University Hospital, Universitat Autònoma de Barcelona, Barcelona, Spain; 11 Clinical Neurosciences, Clinical & Experimental Sciences, Faculty of Medicine, University of Southampton, United Kingdom; 12 National MS Foundation of the Netherlands, Rotterdam, The Netherlands; 13 Department of Community & Occupational Medicine, University Medical Centre Groningen, Groningen, The Netherlands; 14 MS4 Research Institute, Nijmegen, The Netherlands; 15 Institut für Medizinische Biometrie und Epidemiologie, Universitätsklinikum Hamburg-Eppendorf, Hamburg, Germany; Heinrich-Heine-Universitat Dusseldorf, GERMANY

## Abstract

**Background:**

Adequate disease and treatment-related risk knowledge of people with Multiple Sclerosis (pwMS) is a prerequisite for informed choices in medical encounters. Previous work showed that MS risk knowledge is low among pwMS and role preferences are different in Italy and Germany.

**Objective:**

We investigated the level of risk knowledge and role preferences in 8 countries and assessed putative variables associated with risk knowledge.

**Methods:**

An online-survey was performed based on the Risk knowledge questionnaire for people with relapsing-remitting MS (RIKNO 2.0), the electronic Control Preference Scale (eCPS), and other patient questionnaires. Inclusion criteria of participants were: (1) age ≥18 years, (2) a diagnosis of relapsing-remitting MS (RRMS), (3) being in a decision making process for a disease modifying drug.

**Results:**

Of 1939 participants from Germany, Italy, the Netherlands, Serbia, Spain and Turkey, 986 (51%) (mean age 38.6 years [range 18–67], 77% women, 7.8 years of disease duration) completed the RIKNO 2.0, with a mean of 41% correct answers. There were less than 50 participants in the UK and Estonia and data were not analysed. Risk knowledge differed across countries (*p* < 0.001). Variables significantly associated with higher risk knowledge were higher education (*p* < 0.001), previous experience with disease modifying drugs (*p* = 0.001), correct answer to a medical data interpretation question (*p* < 0.001), while higher fear for wheelchair dependency was negatively associated to risk knowledge (*p* = 0.001).

**Conclusion:**

MS risk knowledge was overall low and differed across participating countries. These data indicate that information is an unmet need of most pwMS.

## Introduction

Good clinical practice in medical decision making is increasingly recognised as a shared process between the physician and the patient [1,2]. This so called shared decision making (SDM) is defined as an interactive process between the health care provider (e.g. doctor or nurse) and the patient. Both parties have different and all-relevant information that are essential for the decision making process [3]. In chronic diseases as multiple sclerosis (MS) SDM is even more important, particularly as different treatment options are available [4]. Accordingly, two studies reported that people with MS (pwMS) claim active roles in decision making [4,5].

One prerequisite for SDM is the sharing of relevant disease and treatment-related risk knowledge appraised by methods of evidence-based medicine [6,7], because risk knowledge is necessary to enable patients to get to an informed choice [8]. Risk knowledge as communicated in evidence-based patient information (EBPI) can be measured through questionnaires and can be assessed as a crucial surrogate for SDM processes [9]. It has indeed been shown that EBPI is able to increase informed choices of pwMS [10,11]. In addition, risk knowledge seems to contribute to treatment adherence [10,12].

Previous work indicates low levels of MS risk knowledge among pwMS in Germany [13,14] and differences in role preferences in medical decision making between Germany and Italy [15.]. Until now, little is known about further cross-cultural differences in MS risk knowledge. Thus, the aim of the present study was to investigate the level of risk knowledge amongst pwMS from different European countries, and to assess the role of demographic and clinical characteristics on risk knowledge. In addition, we assessed the role preferences of pwMS regarding medical decision making.

## Material and methods

An online-survey was performed in 8 countries using the secure online survey program EFS Survey.

### Ethics statement

The study protocol was approved by the following: ethics committee of the Hamburg Chamber of Physicians (PV3559 6.9.2010); ethics committee of the Fondazione IRCCS Istituto Neurologico Carlo Besta, Milan, Italy; ethics committee of the Faculty of Medicine University of Belgrade, Serbia; Izmir Katip Celebi University Ethical Review Board, Turkey; Comité Ético de Investigación Clínica del Hospital Universitario Vall d’Hebron, Barcelona, Spain; University of Southampton Ethics and Research Governance Office (approval number: 18783); Medische Ethische Toetsings Commissie Brabant, The Netherlands; Tallinn Medical Research ethics committee, Estonia. Participants were informed that data analysis was anonymous.

### Recruitment of participants

The survey was performed in Estonia, Germany, Italy, the Netherlands, Serbia, Spain, Turkey and the United Kingdom (UK). To get meaningful results per country we aimed to get at least 50 participants from each country. Different recruitment strategies were applied: Germany and Spain recruited via homepages, Italy and the Netherlands via National MS Society mailing lists, Serbia and Turkey via personal contact with patients in the MS clinic.

Inclusion criteria of participants were: (1) age ≥18 years, (2) a diagnosis of relapsing-remitting MS (RRMS) (as the main questionnaire addresses people with RRMS), (3) being in a decision making process for a disease modifying drug (DMD) as we aimed to address those patients for whom risk knowledge is most relevant.

### Questionnaires

The Risk knowledge questionnaire for people with RRMS (RIKNO 2.0) is a self-assessed inventory consisting of 19 multiple choice items; a total score (range 0–21) is obtained by summing up correct answers [13,14]. The RIKNO 2.0 was translated-culturally adapted from German to UK English, and from UK English into Dutch, Estonian, French, Italian, Serbian, Spanish and Turkish using a recognised procedure [14]. All the RIKNO 2.0 versions used in the online survey are reported in S1 Appendix. A German validation study with 708 pwMS showed on average 10.2 correct answers (SD 3.3). Mean item difficulty was 46% (range 20–79%) [14].

The Multiple Sclerosis Knowledge Questionnaire (MSKQ) is a 25-item self-assessed, on general MS knowledge [16], for newly-diagnosed MS patients. Originally developed in Italian, translations of the MSKQ have been performed to Dutch, English, and German. The total score is obtained by summing up correct answers (possible range: 0–25). Two versions of the questionnaire exist, for re-administration (in the present survey we used version A). The MSKQ was well accepted by pwMS, it has good reliability and, importantly, proved to be responsive in a randomised-controlled trial on an information aid for newly-diagnosed pwMS [16].

The electronic, self-administered Control Preference Scale (eCPS) was used to assess the patient preferred interaction style in medical decision making. It consists of five “cards” each describing, by a cartoon and a written sentence, a role preference. Based on the first two preferred cards, participants are classified into 6 role preference styles that can be collapsed into 3 (autonomous, shared, and passive) [15]. The eCPS was found to be well accepted and useful by pwMS; it was as reliable as the original face-to-face version [15]. Originally devised in US English, the CPS was translated-adapted into Italian, German, Serbian, Estonian and Dutch.

### Demographic and clinical data

Participants filled in the following general information: gender, age, education, and living situation. They also completed the following: MS course and duration, current decision making process, current and former DMD treatment, level of impairment (Patient Determined Disease Steps, PDDS scale) [17,18], type of referral center, medical data interpretation competence (single question with 3 possible answers, one correct) [19], perception of disease severity (visual analogue scale, VAS), fear for wheelchair dependency (VAS). [20] General risk aversion/inclination was assessed with a VAS and general risk behaviour (VAS) [21].

Our main focus was the RIKNO 2.0 questionnaire, thus the order of presentation was the following: general and clinical questions, RIKNO 2.0, eCPS, and MSKQ. As some questionnaires were not available in all the nine languages, questionnaires used in each country are listed in S1 Table.

### Hypotheses

The following study questions and hypotheses were investigated: (1) There is an overall low level of risk knowledge and different risk knowledge levels between the participating countries. (2) Concerning variables associated to risk knowledge, we hypothesized people with higher educational background, younger age [21] and living in northern Europe to have higher risk knowledge. In addition, we hypothesized a positive association of risk knowledge with the following variables: gender, current follow-up at a MS center, level of impairment, current DMD treatment, previous experience with DMD, being in a DMD decision making phase, medical data interpretation ability, disease duration, perception of disease severity, and fear for wheelchair dependency. (3) Assuming that MSKQ addresses general MS knowledge in newly-diagnosed pwMS, we hypothesized that RIKNO 2.0 and MSKQ are moderately correlated. (4) Based on previous work, we hypothesized that pwMS of different countries to have different role preferences [15].

The data which form the basis for the analysis can be found in S1 Dataset.

### Data analysis

We assumed on average less than 50% correct answers (total score <10.5) in the RIKNO 2.0. Differences in RIKNO 2.0 scores across countries were calculated using ANOVA. A mixed model regression approach was used to investigate the effect on RIKNO 2.0 scores of the following independent variables: gender, education, being followed at a MS center, being on DMD, previous experience with DMD, medical data interpretation competence, level of impairment, age, disease duration, perception of disease severity, and fear for wheelchair dependency. Country was entered as a cluster variable. RIKNO 2.0 and MSKQ were compared using paired t-tests. Due to different range of values of the sum scores of RIKNO 2.0 (0–21) and MSKQ (0–25), MSKQ-data were fitted to the range of values of RIKNO 2.0. Pearson’s correlation coefficients were used to assess concurrent validity, assuming r ≤ 0.5. Possible differences in role preferences among pwMS of different countries were analyzed using the χ^2^-test. General and clinical features were compared across countries using χ^2^-tests for categorical variables and ANOVA for continuous variables, as appropriate. To estimate sampling bias we checked if participants who finished the whole survey differed to those who discontinued after filling in demographic data using χ^2^-tests for categorical variables and ANOVA for continuous variables.

All *p* values were two tailed, and considered significant at 0.05 level. Analyses were performed using SPSS version 24.0.

## Results

Out of 1939 pwMS who started the survey, 1002 completed the RIKNO 2.0 questionnaire. Participants from Estonia and the UK were <50, and thus were not analysed. Overall, 986 out of 1939 participants from the remaining seven countries (51%) did complete the RIKNO 2.0 questionnaire: completer rates ranged from 93% (Turkey) to 47% (Germany; Table 1).

**Table 1:** General and clinical characteristics of people with multiple sclerosis in the six countries. Characteristics refer to completers of RIKNO 2.0 only.

	Total (N = 1939)	Germany (N = 504)	Italy (N = 208)	Serbia (N = 135)	Spain (N = 706)	Netherlands (N = 169)	Turkey (N = 217)	*P* value
RIKNO 2.0 completers^a^	986 (51)	184 (37)	84 (40)	105 (78)	279 (40)	133 (79)	201 (93)	0.005
Women^b^	755 (77)	142 (77)	78 (93)	77 (74)	213 (76)	123 (93)	122 (61)	<0.001
Age (years)^c^	38.6 (18–67)	38.2 (19–60)	40.6 (26–64)	36.9 (20–57)	40.8(18–67)	40.6 (23–60)	34.6 (18–62)	<0.001
Disease duration (years)^c^	7.8 (0–37)	5.5 (0–28)	7.7 (1–30)	8.5 (0–35)	9.9 (0–37)	4.7 (0–22)	8.5 (0–32)	<0.001
RRMS ^b^^,^^d^	934 (95)	163 (89)	79 (94)	101 (96)	265 (95)	129 (97)	197 (98)	0.001
In a DMD decision making process^b^	764 (77)	184 (100)	56 (67)	105 (100)	148 (53)	70 (53)	201 (100)	<0.001

DMD, disease-modifying drug; RRMS, relapsing-remitting multiple sclerosis; RIKNO, Risk knowledge questionnaire in multiple sclerosis.

^a^ N (% of column total)

^b^ N (% of column RIKNO 2.0 completers)

^c^ Mean (range)

^d^ All other participants were newly diagnosed people with multiple sclerosis.

### General and clinical characteristics

Participants who completed the RIKNO 2.0 were on average 38.58 years old with about 7.75 years of disease duration (Table 1). As expected, about two thirds of participants were women. We found small, but significant differences in a couple of demographic characteristics between different countries (see S2 Table). Most striking patients in Turkey were the youngest. PwMS who did not complete the RIKNO 2.0 were older (mean age 40.64, *p* = 0.004) and fewer were currently on a DMD (64%, *p* = 0.29). Non completers were less often followed at a MS center (34%, *p* < 0.001), and were more inclined to take general risks in life (mean 6.68, *p* = 0.004; with 0 = risk aversive and 10 = risk inclined). In addition they showed a lower frequency of correct answers to the medical data interpretation question (72%, *p* = 0.008). In Italy, Spain and the Netherlands, RIKNO 2.0 scores of pwMS in (*N* = 339, mean 7.20, SD 3.51) and outside of a DMD decision-making process (*N* = 262, mean 6.92, SD 3.36) were not different (*p* = 0.315).

### Risk knowledge

**Fig 1:**
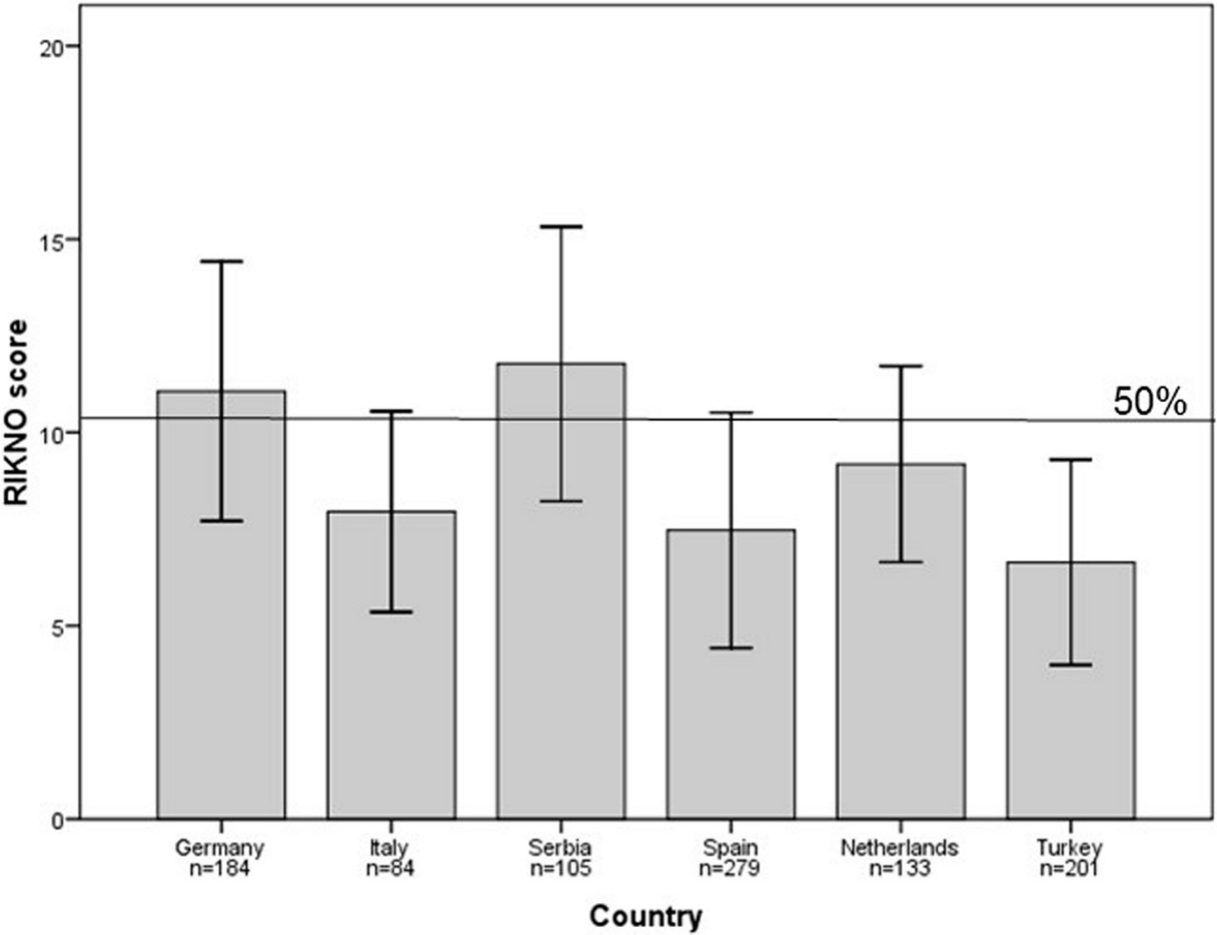
Mean and SD level of multiple sclerosis risk knowledge (RIKNO 2.0) by country. Possible ranges are 0–21.

**Fig 2:**
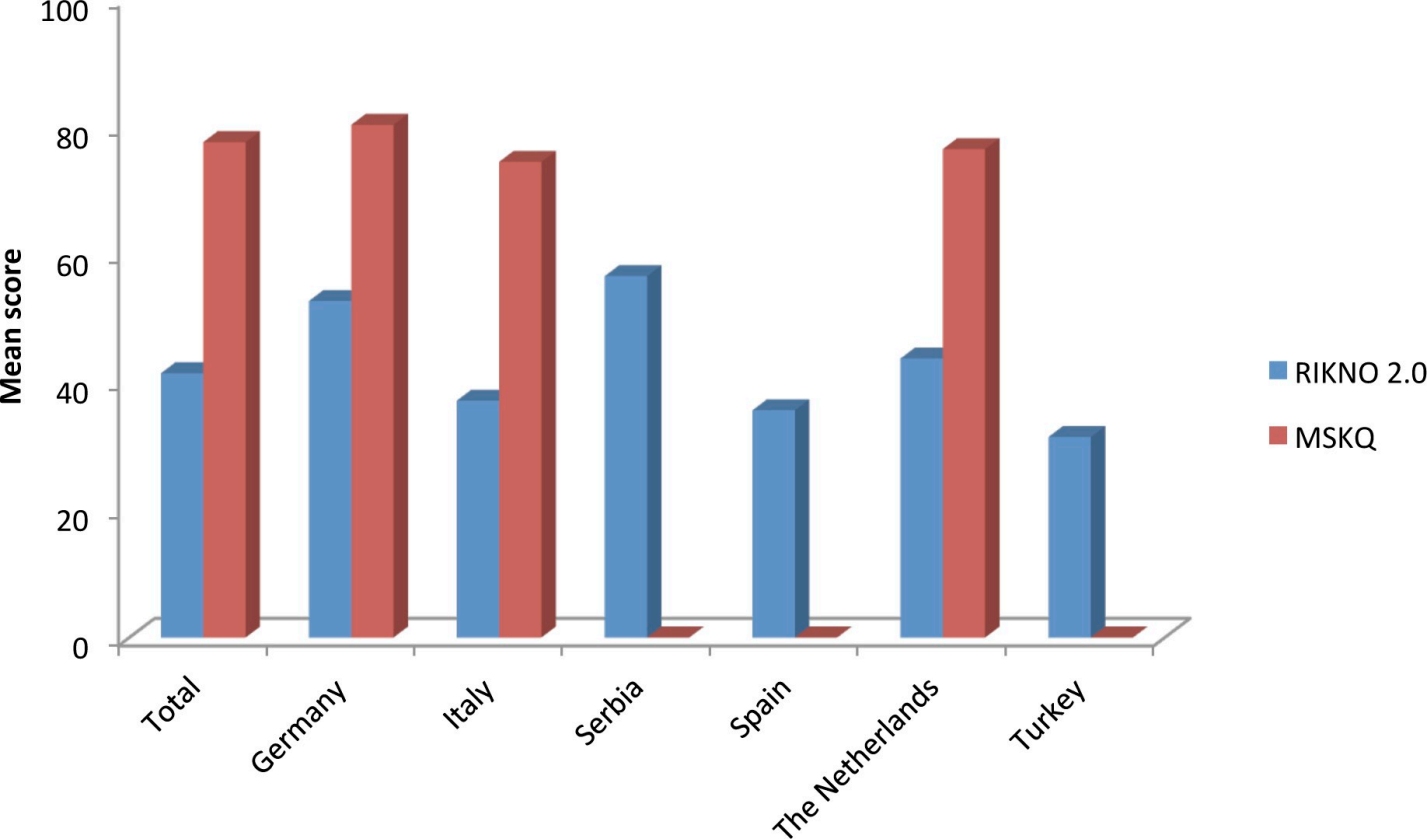
Mean RIKNO 2.0 scores of the PwMS (blue columns) in comparison to the MSKQ (red columns)]. MSKQ, Multiple sclerosis knowledge questionnaire; RIKNO, Risk knowledge questionnaire in multiple sclerosis; SD, standard deviation. For easier comparability we transformed scores to a 0–100 scale.

The mean number of correct answers in RIKNO 2.0 within the whole sample was 8.7 (SD 3.5) out of 21, which means that participants gave on average 41% correct answers (see Figs 1 and 2, and S3 Table for item difficulties). Differences between countries for RIKNO 2.0 scores were significant (*p* <0.001), with similar results for Italy, Spain, the Netherlands and Turkey, and slightly higher scores for Germany and Serbia.

### Variables associated to risk knowledge

Variables significantly associated to RIKNO 2.0 were country (cluster variable), education, previous DMD experience, medical data interpretation ability, and fear for wheelchair dependency (see Table 2). Specifically, non-graduated pwMS provided on average 1.62 less correct answers to RIKNO 2.0 than graduates. PwMS who replied not correctly to the question on medical data interpretation had 1.31 less correct answers than those who replied correctly. PwMS with no previous DMD experience had on average about 0.87 less correct answers than those who had been treated with DMDs. Most pwMS of all countries had high ratings of fear for wheelchair dependency (S2 Table). Participants with maximum fear for wheelchair dependency had less correct answers in the RIKNO 2.0 than people with the lowest fear. No association of age with risk knowledge could be shown.

**Table 2:** Variables associated to RIKNO 2.0 score (linear mixed regression model). Significant values are reported in bold.

Parameter	Estimate (score)	SE	95% CI	*p* value
Country (cluster variable)				**<0.001**
Intercept	12.32	1.27		
Women	0.17	0.27	-0.71 to 0.35	0.52
Education: not graduated	-1.62	0.24	-2.05 to 1.12	**<0.001**
Not followed at MS center	-0.16	0.29	-0.78 to -0.37	0.60
No current DMD treatment	0.41	0.48	-0.52 to 1.36	0.39
DMD naive	-0.87	0.26	-1.36 to -0.31	**0.001**
Wrong medical data interpretation	-1.31	0.28	-1.88 to -0.77	**<0.001**
Impairment level (PDDS)	-0.15	0.08		0.06
Age (years)	-0.01	0.02	-0.03 to 0.03	0.70
Disease duration (years)	0.03	0.02	-0.01 to 0.07	0.17
Perception of MS severity^a^	0.01	0.01	-0.01 to 0.03	0.33
Fear for wheelchair dependency^b^	-0.04	0.01	-0.06 to -0.01	**0.001**

CI, confidence interval; DMD, disease modifying drug; MS, multiple sclerosis; PDDS, patient determined disease steps; SE, standard error.

^a^ Visual analogue scale: 1 = benign, 51 = severe.

^b^ Visual analogue scale: 1 = no problem, 51 = worst thing imaginable.

### Comparison of RIKNO 2.0 and MSKQ

Within the subsample with RIKNO 2.0 and MSKQ (*N* = 294) adjusted mean RIKNO 2.0 score was 41.4 (SD 16.7) and mean MSKQ score was 77.6 (SD 11.8). The paired t-test showed a significant mean score difference between the inventories (mean difference = 36.2, *p* <0.01): participants got higher scores with the MSKQ. However, correlation between MSKQ and RIKNO 2.0 was moderate (*r* = 0.480, *p* <0.001).

### Control preferences

**Fig 3:**
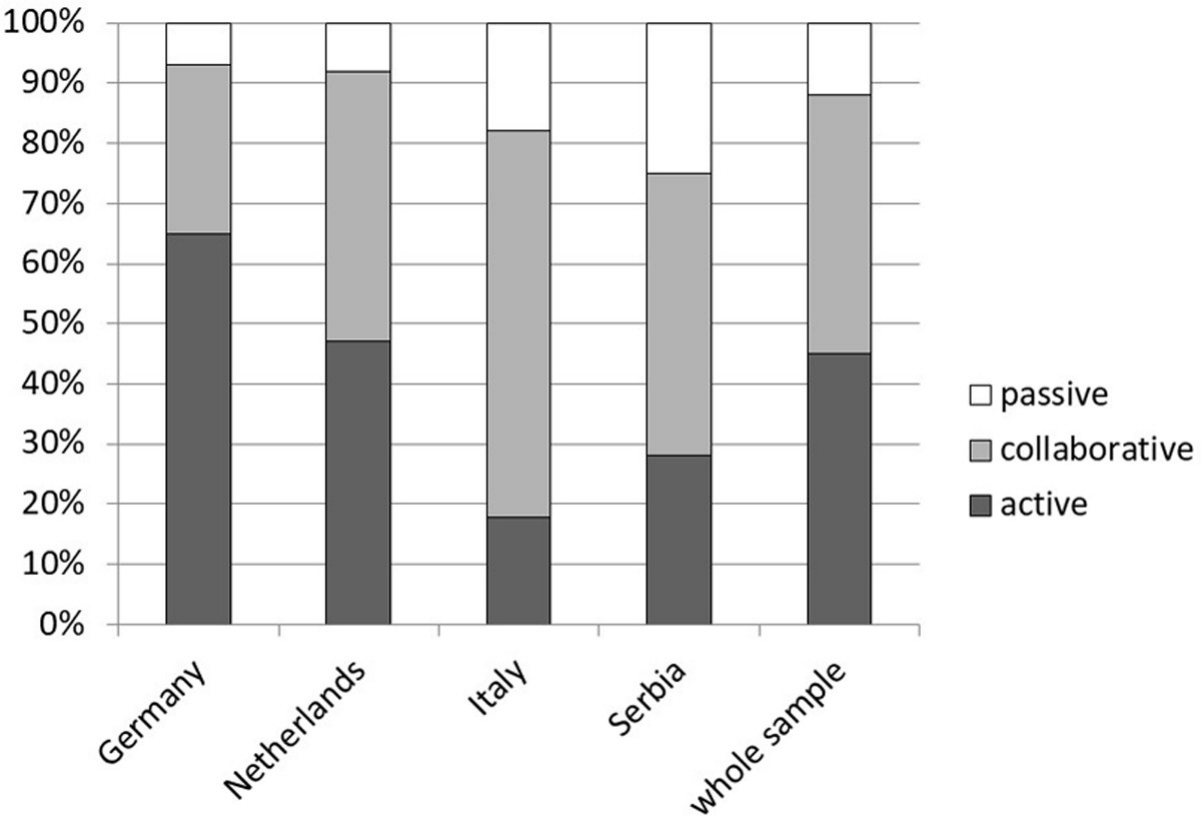
Distribution of role preferences of people with multiple sclerosis in four countries (*N* = 364) assessed with the electronic Control Preference Scale (eCPS).

Overall, participants who completed the eCPS (*N* = 364), expressed more active and collaborative role preferences than passive (Fig 3 and S2 Appendix). More than half of the German and Dutch pwMS preferred an active role (*p* <0.001), while half of the Italian and Serbian participants preferred a collaborative role, followed by passive and active roles.

## Discussion

This survey is to our knowledge the largest investigation on risk knowledge of pwMS with most of our hypotheses supported by the results of the study.

First, overall risk knowledge was low in all the countries. Germany and Serbia showed best results, but with only about 52% correct answers. Risk knowledge is a prerequisite for informed choice [9] and it is essential for SDM. If patients do not have enough risk knowledge, they are not able to really participate in decision making processes. Thus, there seems to be a need for improved MS information material and programs to enhance DMD risk knowledge [10,22]. However, there is no standard on how to define a set of relevant risk knowledge data. The RIKNO 2.0 questionnaire presented here was originally devised more than a decade ago, with a first tool published in 2004 [5], and was further revised and updated through the AutoMS project [13,14] with input from MS experts and pwMS across Europe. This revision process was necessary to harmonize RIKNO 2.0 contents across different cultures, and to incorporate new evidence especially on DMDs.

A second finding was that MS risk knowledge varied among countries, similarly to what we found in role preferences using the eCPS [15]. The heterogeneity in risk knowledge can be at least in part due to differing recruitment strategies: In Turkey and Serbia participants were recruited during face-to-face encounters at the MS center, leading to a higher response rate. This may as well have led to the younger age of the Turkish cohort. In the Netherlands and Italy recruitment was via the National MS Society mailing list, and in Germany via the homepage of the German MS Self-Help Society. However, focusing the two centers with face-to-face recruitment one had the highest (Serbia, mean RIKNO 2.0 total score 57) and the other the lowest (Turkey, mean score 31) knowledge. Therefore a strong effect of recruitment strategy on knowledge seems unlikely. Similarly, the remaining four centers, all with remote recruitment, had variable RIKNO 2.0 scores, ranging from 58 in Germany to 36 in Spain. From these groups homogeneous in sampling approach, a geographic effect manifested, with lower risk knowledge in countries from the Mediterranean Europe (Spain, Italy, and Turkey) vs. the other (Germany, the Netherlands, and Serbia). As well the minor differences in the distribution of demographic factors cannot explain this pattern. Being within or outside of a decision making process seemed also not to influence the knowledge level. In the whole sample 50% of patients were graduated and only 60% followed at MS centers which we believe is representative for an MS cohort in the Western world and does not mirror a highly educated and high MS care integrated sample.

Patients who did not complete the RIKNO 2.0 were slightly older, less often followed at an MS centers and had lower medical data interpretation abilities. As expected, most drop-outs were generated in countries that recruited via homepages and mailing lists.

Lower medical data interpretation ability and education were associated with lower RIKNO 2.0 scores. Interestingly, previous DMD experience was associated with higher risk knowledge (*p* <0.01), which can be due to direct DMD experience and learning, and to an increased personal relevance of RIKNO 2.0 contents. In fact patients on a DMD might have received more risk information from their doctors than those refraining from treatments. Most participants had high-level fear for wheelchair dependency with little variation across countries, with a weak but significant negatively association with risk knowledge. It is possible that a higher wheelchair dependency fear may lead to denial and information blocking behavior leading to less up-to-date information. Therefore psychological factors need to be taken into account when studying risk knowledge and educational interventions.

Fourth, the comparison of RIKNO 2.0 and MSKQ showed expected results, too, with the two inventories being moderately correlated. They measure similar domains, but with different aims, as the MSKQ appraises general MS knowledge, while RIKNO 2.0 is a more demanding tool focused on MS treatments.

Finally, the eCPS showed different role preference distributions in the four countries, with German and Dutch pwMS having more active role preferences than pwMS from Italy and Serbia. These findings confirm and expand previous results using this scale in pwMS. [15] Hence, the difference in interaction style preferences can be due to cultural and social characteristics, or to the different health systems of these European countries. Taken together the lower knowledge level in Mediterranean countries with a lower level of autonomy preference might indicate a higher paternalistic role understanding between patients and physicians in these countries. Admittedly, different sampling methods might have also contributed to these differences. There is a need for further studies, with qualitative methods, to further investigate this important topic.

This study has some limitations. In Italy, it was necessary to have three rounds of invitation (on a random sample of 600, 1000, and 600 pwMS registered to the Italian National MS Society mailing list) to achieve the target number of participants, and for the third round the enrolment criterion of being in a DMD decision-making process was dropped. Recruitment strategies of UK and Estonia were not successful. In the UK, 183 eligible patients were offered recruitment by post, and only 19 completed it. Telephone calls with non-responders indicated that they found the questionnaire too difficult. Another limitation was that some questionnaires/inventories were not administered in all participating countries, so for example the eCPS could not be included in the RIKNO multivariate model. Difficulties encountered during the development and refinement of a risk knowledge tool have been elucidated in a recent publication [14].

## Conclusions

The survey revealed an overall low MS risk knowledge level among pwMS in Europe. Since well informed decision by patients is not possible without relevant medical knowledge, these results are highly relevant, confirming the need for improvements in information provision. In addition, our findings indicate that cross-cultural differences exist not only in role preferences, but also in MS risk knowledge. Responding to the specific information needs of patients is key, and RIKNO 2.0 can be further proposed as a valid outcome measure.

## Supporting information

S1 Appendix Risk knowledge questionnaire for people with relapsing-remitting MS (RIKNO 2.0). (DOCX)

S2 Appendix Electronic Control Preference Scale distribution by country. (DOCX)

S1 Table Questionnaires used in each country. (DOCX)

S2 Table Characteristics of participants by country. (DOCX)

S3 Table Item characteristics of the RIKNO 2.0 and MSKQ. (DOCX)

S1 Dataset Raw data from patients’ general and clinical characteristics, Multiple Sclerosis Knowledge Questionnaire (MSKQ), Risk knowledge questionnaire for people with relapsing-remitting MS (RIKNO 2.0), electronic Control Preference Scale (eCPS). (SAV)
