# Supplementary material for: Risk knowledge of people with relapsing-remitting multiple sclerosis – Results of an international survey
Source: PLoS One. 2018 Nov 29;13(11):e0208004. doi: 10.1371/journal.pone.0208004 (PMC6264873; doi:10.1371/journal.pone.0208004)
Supplement: S1 Appendix — (DOCX) [file pone.0208004.s001.docx]

**S1 Appendix.** The RIKNO 2.0 Questionnaire, Ver. 1.1.2015, is reported in the following versions: English (pp 1-5), German (pp 6-10), Italian (pp 11-15), Serbian (pp 16-20), Spanish (pp 21-25), Dutch (pp 26-31), and Turkish (pp 32-35). For each question, the correct answer is underlined.

**___________________________________________________________________________**

This questionnaire assesses your knowledge of MS. It was especially developed to assess risk knowledge in education programmes for patients who are considering immunotherapy. You might have difficulty answering the questions. But bear in mind: it is not meant as a test to judge whether you are "good" or "bad".

It was developed rather as an indicator for health professionals to gauge the need to deliver support when informing you about the disease.

Please read each question and tick only one answer.

Please read the following questions and tick the one answer in each group that you consider to be correct.

1. **Which one of the following statements about relapses is correct?**

- Frequent relapses predict a faster worsening of impairment in the future, whenever they occur in the course of MS.
- Frequent relapses during the first 2 years of the disease predict a faster worsening of impairment in the future.
- If there are fewer relapses during the course of the disease it is a sign of improvement of the disease.
- Severe relapses in any disease phase point to a faster worsening of impairment in the future.

1. **Which one of the following statements about MRI is correct?**

- A patient’s impairment can be determined by the MR image.
- Contrast medium (gadolinium) enhancement visible on the MRI is a sign of active inflammation.
- All MS patients should have a MRI done at least once a year.
- Inflamed areas on the MRI (white spots) indicate destruction of nerve sheaths and nerve cells.

1. **Which one of the following statements about MS types is correct?**

- When a MS diagnosis is made the disease course can be determined as well.
- The switch from a relapsing to a chronic progressive course can be determined only in retrospect.
- Very few MS patients have a relapsing MS course from onset.
- The disease course of MS is not relevant for treatment decisions.

1. **Which one of the following statements about MS types is correct?**

- Up to 30 out of 100 MS patients will remain without relevant impairments even after 20 years with MS (benign MS).
- There is no such thing as benign MS.
- Sooner or later all MS patients develop clearly noticeable, lasting impairments.
- The development of impairments in MS does not depend on the disease type (relapsing or progressive).

1. **Which one of the following statements about the long-term course of MS is correct?**Studies carried out on MS patients who have never received immunotherapy show that after 15 years walking ability was almost unrestricted in…

- approximately 90 out of 100 patients.
- approximately 70 out of 100 patients.
- approximately 50 out of 100 patients.
- approximately 20 out of 100 patients.

1. **Which one of the following statements about the EDSS scale is correct?**

- From the EDSS scale one can identify all relevant impairments in a patient.
- EDSS scores from 4.0 to 7.0 are predominantly determined by the distance that patients can walk.
- Increases in impairments are always reflected by increases in the EDSS.
- Changes in vision and cognitive ability are assessed well by the EDSS scale.

1. **Which one of the following statements is correct?**A double-blind, randomised, placebo-controlled trial is…

- a trial in which a new drug is tested against an old one.
- a trial in which a drug is tested against a dummy drug (placebo). The patients are randomly assigned to receive the drug or the placebo. Neither doctor nor patient knows who gets which substance.
- a trial in which a drug is tested against a placebo. The patients are randomly assigned to receive the drug or the placebo. The study physicians are informed about which substance is given to their patients.
- a trial in which patients with their eyes blindfolded twice try out different drugs.

1. **The following three questions concern stability of impairment in trials on interferons in MS (8a, 8b, 8c). Please answer all three questions.**
2. **Which of the following statements on the results of placebo treatment is correct?**Stability of MS when taking placebo:
   Imagine 100 patients with relapsing MS who all have 2 relapses per year. How many of these 100 patients can expect their level of impairment to remain stable on treatment with placebo within the next 2 years?

- about 15 of those 100
- about 25 of those 100
- about 40 of those 100
- about 70 of those 100
- about 80 of those 100

1. **Which one of the following statements about the results of interferon treatment is correct?**Stability of MS when taking interferon:
   Imagine 100 patients with relapsing MS who all have 2 relapses per year. How many of these 100 patients can expect their level of impairment to remain stable on treatment with interferon within the next 2 years?

- about 15 of those 100
- about 25 of those 100
- about 40 of those 100
- about 70 of those 100
- about 80 of those 100

1. **Which one of the following statements about treatment with interferon versus placebo is correct?**In answer to question 8a you stated how many of the 100 patients will remain stable without therapy within the next 2 years. Now, how many **more** patients will remain stable as a result of interferon therapy (i.e. **in addition** to those who will remain stable without therapy)?

- about 10 of those 100
- about 25 of those 100
- about 45 of those 100
- about 55 of those 100
- about 65 of those 100

1. **Which one of the following statements about therapy of different MS types is correct?**

- There are currently no trials proving the effectiveness of treatments for patients with the first signs and symptoms of MS.
- There are currently no trials proving the effectiveness of treatments for patients with relapsing MS.
- There are currently no trials proving the effectiveness of treatments for patients with secondary progressive MS.
- There are currently no trials proving the effectiveness of treatments for patients with primary progressive MS.

1. **Which one of the following statements concerning Copaxone**^®^ **(Glatiramer acetate) is correct?**

- The effectiveness of Copaxone^®^ for reducing relapse rates is comparable to that of interferons.
- The effectiveness of Copaxone^®^ for reducing relapse rates is better than that of interferons
- The effectiveness of Copaxone^®^ for reducing relapse rates is worse than that of interferons.
- It is not possible to compare the effectiveness of Copaxone^®^ and interferons for reducing relapse rates.

1. **Which one of the following statements about flu-like symptoms, a side-effect of interferons, is correct?**

- All MS patients will experience flu-like symptoms at least once at some stage of interferon treatment.
- Flu-like symptoms appear only at the beginning of interferon treatment.
- Approximately 50 out of 100 MS patients will experience flu-like symptoms at the beginning of interferon treatment.
- Approximately 10 out of 100 MS patients will experience flu-like symptoms at the beginning of interferon treatment.

1. **Which one of the following statements about MS therapies with tablets is correct?**

- Gilenya^®^ (Fingolimod) is more effective than Tysabri® (Natalizumab).
- Gilenya^®^ (Fingolimod) has hardly any side-effects.
- The efficacy of Tecfidera^®^ (Dimethylfumarat) is similar to that of the interferons.
- Aubagio^®^ (Teriflunomide) is more effective than interferons.

1. **Which one of the following statements about Tysabri^®^ (Natalizumab) is correct?**

- If treated with Tysabri^®^ for 2 years approximately 40 out of 100 patients with relapsing MS do not have an increase in impairment because of the drug.
- More than 50 out of 100 patients suffer side-effects that come on suddenly as a result of treatment with Tysabri^®^.
- Approximately 3 out of 1000 patients who are treated with Tysabri^®^ suffer a severe viral brain infection (Progressive Multifocal Leukoencephalopathy/PML).
- In addition to interferons, Tysabri^®^ is a first-choice treatment for secondary progressive MS.

1. **Which of the following statements concerning pregnancy and MS are correct?**

- The relapse rate increases during pregnancy.
- Contraception is mandatory during any MS treatment.
- One out of 50 children with one parent affected by MS will get the disease as well.
- Breast feeding increases the risk of relapses.

1. **Which of the following statements concerning complementary medicine and nutritional supplements is correct?**

- Studies have shown that enzyme therapy reduces relapse rates.
- Studies have shown that polyunsaturated acids (e.g. fish oil, evening primrose oil) slow down disease progression.
- There are no convincing studies showing that complementary medicine or nutritional supplements influence the disease activity in MS.
- Studies have shown that vitamin D reduces relapse rates.

**Now, for the following statements please tick the one answer that you consider to be wrong.**

1. **Which one of the following statements concerning diagnosis is wrong?**

- MS diagnosis can be confirmed if typical symptoms and characteristic MRI findings are seen simultaneously and a further MRI made shortly afterwards provides evidence of new inflamed areas.
- In most cases MS can only be diagnosed after the disease has run its course for a while.
- Sometimes it can be difficult to diagnose MS beyond all doubt.
- MS can be diagnosed solely on the basis of antibodies in the cerebrospinal fluid that are only found in MS.

1. **Which one of the following statements about relapses is wrong?**

- Relapses are new symptoms that develop within days or weeks.
- Relapses are old symptoms that flare up for only a few hours.
- Relapses are intensified old symptoms or new symptoms that last for at least 24 hours.
- It can sometimes be difficult to distinguish relapses from daily fluctuations in MS symptoms.

1. **Which one of the following statements about MS therapies is wrong?**

- MS therapies aim to cure the disease.
- MS therapies work best in cases of relapsing MS.
- MS therapies can slow down disease progression.
- MS therapies can reduce the frequency of relapses.

1. **Which one of the following statements about drugs for immunotherapy is wrong?**

- Mitoxantrone may be used for the treatment of relapsing MS.
- Some interferon medications have been approved for the treatment of secondary progressive MS.
- Tysabri^®^ (Natalizumab) has been approved for the treatment of relapsing MS.
- Gilenya^®^ (Fingolimod) has been approved for the treatment of primary and secondary MS.

**Many thanks for your help!**

Der folgende Fragebogen behandelt spezielles Wissen zu MS. Der Bogen dient vor allem zur Überprüfung von Schulungsprogrammen, als Erfolgskontrolle für Ärzte und MS-Schwestern, ob Ihnen alle wichtigen Inhalte vermittelt wurden. Deshalb werden Sie die Fragen möglicherweise schwierig finden. Bitte behalten Sie daher im Hinterkopf, dass es sich nicht um einen Test handelt, ob sie „gut“ oder „schlecht“ sind.

Bitte lesen Sie jede Frage und kreuzen Sie nur eine Antwort an.

Bei den folgenden Fragen kreuzen Sie bitte die eine Antwort an, die Sie für RICHTIG halten.

**1. Welche der folgenden Aussagen über Schübe ist richtig?**

- Häufig auftretende Schübe weisen unabhängig vom Auftreten im Krankheitsverlauf auf eine schnellere Zunahme der Beeinträchtigung in der Zukunft hin.
- Häufig auftretende Schübe in den ersten 2 Krankheitsjahren weisen auf eine schnellere Zunahme der Beeinträchtigung in der Zukunft hin.
- Wenn im MS-Verlauf weniger Schübe auftreten, ist das ein Zeichen für eine Besserung der Erkrankung.
- Schwere Schübe weisen auf eine schnellere Zunahme der Beeinträchtigung in der Zukunft hin, unabhängig vom Auftreten im Verlauf der MS.

**2. Welche der folgenden Aussagen über das Kernspin ist richtig?**

- Am Kernspinbild kann man ablesen, wie stark ein Patient beeinträchtigt ist.
- Kontrastmittelanreicherungen (Gadolinium) im Kernspinbild sind Zeichen einer akuten Entzündung.
- Alle MS-Patienten sollten mindestens einmal jährlich im Kernspin untersucht werden.
- Entzündungsherde (weiße Flecken) im Kernspinbild zeigen die Zerstörung von Nervenscheiden und Nervenzellen.

**3. Welche der folgenden Aussagen über MS-Verlaufsformen ist richtig?**

- Wenn eine MS-Diagnose gestellt wird, kann auch die Verlaufsform bestimmt werden.
- Grundsätzlich kann der Wechsel vom schubförmigen in einen schleichenden Verlauf nur im Nachhinein bestimmt werden.
- Wenige MS-Patienten haben von Anfang an einen schubförmigen Verlauf.
- Die Verlaufsform ist nicht wichtig für die Therapieentscheidung.

**4. Welche der folgenden Aussagen über MS-Verläufe ist richtig?**

- Bis zu 30 von 100 MS-Patienten sind auch nach 20 Jahren mit MS in ihren Alltagsaktivitäten kaum eingeschränkt (gutartiger Verlauf).
- Es gibt keinen gutartigen MS-Verlauf.
- Bei MS entwickeln sich früher oder später immer deutlich spürbare, bleibende Beeinträchtigungen.
- Die Entwicklung von Beeinträchtigungen bei MS hängt nicht von der Verlaufsform ab (schubförmige oder chronisch-progrediente MS).

**5. Welche der folgenden Aussagen über den MS-Verlauf ist richtig?**

Studien mit MS-Patienten, die nie eine Immuntherapie erhalten hatten, zeigen, dass die Gehfähigkeit nach 15 Jahren fast uneingeschränkt war bei…

- ….etwa 90 von 100 Patienten
- ….etwa 70 von 100 Patienten
- ….etwa 50 von 100 Patienten
- ….etwa 20 von 100 Patienten

**6. Welche der folgenden Aussagen über die EDSS-Skala** **ist richtig?**

- Durch den Wert der EDSS-Skala lassen sich alle bedeutsamen Beeinträchtigungen bei einem Patienten identifizieren.
- Der EDSS-Wert wird im Bereich 4,0-7,0 maßgeblich von der Gehstrecke bestimmt.
- Eine Zunahme der Beeinträchtigung spiegelt sich stets in einer Zunahme des EDSS-Wertes wider.
- Veränderungen des Sehens und des Denkens werden von der EDSS-Skala gut erfasst.

**7. Welche der folgenden Aussagen über Studien ist richtig?**

Eine doppelblinde, randomisierte, plazebo-kontrollierte Studie ist …

- … eine Untersuchung, in der ein neues Medikament gegen ein altes geprüft wird.
- … eine Untersuchung, in der ein Medikament (Verum) gegen ein Scheinmedikament (Plazebo) geprüft wird. Die Patienten werden nach dem Zufallsprinzip der Plazebogruppe oder der Verumgruppe zugeteilt. Weder Arzt noch Patient wissen, wer welche Substanz bekommt.
- … eine Untersuchung, in der ein Medikament gegen ein Scheinmedikament geprüft wird. Die Patienten werden nach dem Zufallsprinzip der Plazebogruppe oder der Verumgruppe zugeteilt. Die Studienärzte wissen, welche Substanz die Patienten erhalten.
- … eine Untersuchung, bei der Patienten mit zweifach verbundenen Augen verschiedene Medikamente ausprobieren.

**8. Die folgenden Fragen betreffen die Stabilität der Beeinträchtigung in Studien mit**

**Interferonen (8a, 8b, 8c). Bitte beantworten Sie alle drei Fragen.**

1. **Welche der folgenden Aussagen zu Ergebnissen unter Behandlung mit Plazebo**

**ist richtig?**

Stabilität von MS unter Placebobehandlung:

Stellen Sie sich 100 Patienten mit schubförmiger MS und 2 Schüben pro Jahr vor. Wie viele dieser 100 Patienten können damit rechnen, dass ihr Zustand unter Plazebobehandlung innerhalb der nächsten 2 Jahre stabil bleibt?

• ca. 15 von 100

• ca. 25 von 100

• ca. 40 von 100

• ca. 70 von 100

• ca. 80 von 100

1. **Welche der folgenden Aussagen zu Ergebnissen unter Behandlung mit Interferon ist richtig?**

Stabilität von MS unter Interferon: Stellen Sie sich 100 Patienten mit schubförmiger MS und 2 Schüben pro Jahr vor. Wie viele dieser 100 Patienten können damit rechnen, dass ihr Zustand unter Interferon innerhalb der nächsten 2 Jahre stabil bleibt?

• ca. 15 von 100

• ca. 25 von 100

• ca. 40 von 100

• ca. 70 von 100

• ca. 80 von 100

1. **Welche der folgenden Aussagen zu Vergleich von Interferon- und Plazebobehandlung ist richtig?**

Anders gefragt: Bei Frage 8a haben Sie angegeben, bei wie vielen der 100 Patienten der Zustand ohne Therapie innerhalb der nächsten 2 Jahre stabil bleibt. Wie viele Patienten bleiben durch eine Interferontherapie zusätzlich stabil (z.B. **zusätzlich zu** denjenigen, die auch ohne Therapie stabil bleiben)?

• ca. 10 von 100

• ca. 25 von 100

• ca. 45 von 100

• ca. 55 von 100

• ca. 65 von 100

**9. Welche der folgenden Aussagen zur Therapie verschiedener MS-Verläufe ist richtig?**

- Es gibt bislang keine überzeugenden Therapiestudien mit Wirksamkeitsnachweis für die Behandlung von Patienten mit Erstmanifestation der MS.
- Es gibt bislang keine überzeugenden Therapiestudien mit Wirksamkeitsnachweis für die Behandlung von Patienten mit schubförmiger MS.
- Es gibt bislang keine überzeugenden Therapiestudien mit Wirksamkeitsnachweis für die Behandlung von Patienten mit sekundär-progredienter MS.
- Es gibt bislang keine überzeugenden Therapiestudien mit Wirksamkeitsnachweis für die Behandlung von Patienten mit primär-progredienter MS.

**10. Welche der folgenden Aussagen über Copaxone® (Glatiramer acetat) ist richtig?**

- Die Wirkung von Copaxone® auf die Senkung der Schubrate ist vergleichbar mit der von Interferonen.
- Die Wirkung von Copaxone® auf die Senkung der Schubrate ist besser als die von Interferonen.
- Die Wirkung von Copaxone® auf die Senkung der Schubrate ist geringer als die von Interferonen.
- Die Wirkungen von Copaxone® und Interferonen auf die Senkung der Schubrate sind nicht vergleichbar.

**11. Welche der folgenden Aussagen über grippeähnliche Symptome, eine**

**Nebenwirkung von Interferonen ist richtig?**

- Alle MS-Patienten, die eine Interferon-Behandlung durchführen, haben irgendwann während des Behandlungsverlaufs mindestens einmal grippeähnliche Symptome.
- Grippeähnliche Symptome treten nur zu Beginn einer Interferontherapie auf.
- Etwa 50 von 100 MS-Patienten haben zu Beginn der Interferon-Behandlung grippeähnliche Symptome.
- Etwa 10 von 100 MS-Patienten haben zu Beginn der Interferon-Behandlung grippeähnliche Symptome.

**12. Welche der folgenden Aussagen über MS-Therapien mit Tabletten ist richtig?**

- Gilenya® (Fingolimod) ist wirksamer als Tysabri (Natalizumab).
- Gilenya® (Fingolimod) verursacht kaum Nebenwirkungen.
- Tecfidera® (Dimethylfumarat) ist ähnlich wirksam wie Interferone.
- Aubagio® (Terifunomid) ist wirksamer als Interferone.

**13. Welche der folgenden Aussagen über Tysabri® (Natalizumab) ist richtig?**

- Eine Tysabribehandlung über 2 Jahre verhindert bei etwa 40 von 100 Patienten mit schubförmigem Verlauf eine Zunahme der Beeinträchtigung.
- Bei mehr als 50 von 100 Patienten führt die Therapie mit Tysabri® zu akuten Nebenwirkungen.
- Bei ungefähr 3 von 1000 Patienten, die mit Tysabri® behandelt werden, tritt eine schwere erregerbedingte Hirnentzündung auf (Progressive Multifokale Leukoenzephalopathie/PML).
- Neben Interferonen ist Tysabri® das Mittel der Wahl bei sekundär-progredienter MS.

**14. Welche der folgenden Aussagen zur Schwangerschaft bei MS ist richtig?**

- In der Schwangerschaft steigt die Krankheitsaktivität.
- Bei jedem MS-Medikament muss konsequent Verhütung betrieben werden.
- Eins von 50 Kindern, bei denen ein Elternteil MS hat, wird ebenso an MS erkranken.
- Stillen erhöht das Schubrisiko.

**15. Welche der folgenden Aussagen zum Wirksamkeitsnachweis von**

**Alternativmedizin und Nahrungsergänzung bei MS ist richtig?**

- Studien haben gezeigt, dass eine Enzymtherapie die Schubrate senkt.
- Studien haben gezeigt, dass mehrfach ungesättigte Fettsäuren (z.B. Fischöl, Nachtkerzenöl) das Fortschreiten der Erkrankung verlangsamen.
- Bis heute liegen keine überzeugenden Studien zur Wirksamkeit von Alternativmedizin oder Nahrungsergänzung zur Beeinflussung der Krankheitsaktivität vor.
- Studien haben gezeigt, dass Vitamin D die Schubrate senkt.

**Bei den folgenden Fragen kreuzen Sie bitte die eine Antwort an, die Sie für falsch halten.**

**16. Welche der folgenden Aussagen über die Diagnosestellung ist falsch?**

- Eine MS-Diagnose kann als gesichert gelten, wenn typische Beschwerden und charakteristische Kernspin-Befunde zeitgleich festgestellt und kurzfristig in einem weiteren Kernspin neue Entzündungsherde nachgewiesen werden.
- Oft kann eine MS-Diagnose nur gestellt werden wenn die Erkrankung bereits einige Zeit bestanden hat.
- Im Einzelfall kann es schwierig sein, eine MS-Diagnose zweifelsfrei zu stellen.
- Im Nervenwasser lassen sich MS-typische Antikörper nachweisen, mit denen ohne weitere Untersuchungen die Diagnose gestellt werden kann.

**17. Welche der folgenden Aussagen über Schübe ist falsch?**

- Schübe sind neue Beschwerden, die sich innerhalb von Tagen oder Wochen entwickeln.
- Schübe sind alte Beschwerden, die eventuell nur für einige Stunden wieder auftreten.
- Schübe sind verstärkte alte oder neu auftretende Beschwerden, die mindestens 24 Stunden lang anhalten.
- Manchmal kann es schwierig sein, Schübe von täglichen Schwankungen, den sogenannten Fluktuationen, abzugrenzen.

**18. Welche der folgenden Aussagen über MS-Therapien ist falsch?**

- MS-Therapien zielen auf eine Heilung der Krankheit ab.
- MS-Therapien wirken am besten bei schubförmiger MS.
- MS-Therapien können eine Verlangsamung des Krankheitsprozesses bewirken.
- MS-Therapien können eine Verminderung der Schubrate bewirken.

**19. Welche der folgenden Aussagen über Medikamente zur Immuntherapie ist falsch?**

- Mitoxantron ist zur Behandlung der schubförmigen MS zugelassen.
- Einige Interferone sind zur Behandlung der sekundär progredienten MS zugelassen.
- Tysabri® (Natalizumab) ist zur Behandlung der schubförmigen MS zugelassen.
- Gilenya® (Fingolimod) ist zur Behandlung der primär und sekundär progredienten MS zugelassen.

**Vielen Dank für Ihre Teilnahme!**

Il presente questionario ha lo scopo di valutare la sua conoscenza della sclerosi multipla (SM). Il questionario è stato messo a punto per valutare la conoscenza del rischio nei percorsi di formazione rivolti alle persone con SM che stanno valutando di iniziare o cambiare la terapia immunomodulante. É possibile che lei trovi i contenuti del questionario difficili. Vogliamo tuttavia sottolineare che i risultati della sua compilazione non saranno impiegati per emettere un “giudizio” su di lei, bensì per migliorare il trasferimento delle informazioni sulla malattia da parte del personale sanitario.

Per favore, legga ciascuna domanda indicando con un segno una sola risposta tra quelle riportate.

Per le domande seguenti indichi la risposta che ritiene corretta

1. **Quale delle seguenti affermazioni sulle ricadute è corretta?**

- Ricadute frequenti indicano un più veloce aumento dei deficit neurologici nel futuro, in qualsiasi momento essi si verifichino nel corso della SM.
- Ricadute frequenti nei primi 2 anni della malattia indicano un più veloce aumento dei deficit neurologici nel futuro.
- Se il numero di ricadute diminuisce, a questo corrisponde un miglioramento della malattia.
- Alle ricadute gravi, in qualsiasi fase di malattia, corrisponde un più rapido peggioramento dei deficit neurologici nel futuro.

1. **Quale delle seguenti affermazioni sulla risonanza magnetica nucleare (RMN) è corretta?**

- Dal risultato della RMN è possibile determinare il grado di deficit neurologico del paziente.
- L’aumento del segnale visibile alla RMN, dovuto alla presa del mezzo di contrasto (gadolinio), indica la presenza di infiammazione.
- Tutte le persone con SM dovrebbero fare la RMN almeno una volta all’anno.
- Le aree di infiammazione alla RMN (“macchie bianche”) indicano la distruzione della guaina nervosa e delle cellule nervose.

1. **Quale delle seguenti affermazioni sulle forme di SM è corretta?**

- La forma di SM può essere determinata già al momento della diagnosi di malattia.
- Il passaggio dalla forma recidivante-remittente (a ricadute) a quella progressiva può essere determinatao solo a posteriori.
- Sono molto poche le persone con SM che hanno una forma recidivante-remittente (a ricadute) sin dall’inizio.
- La forma di SM non è rilevante nella scelta della terapia.

1. **Quale delle seguenti affermazioni sulle forme di SM è corretta?**

- Su 100 persone con SM 30 rimarranno in assenza di deficit neurologici rilevanti anche dopo 20 anni di malattia (SM benigna).
- Non esiste una SM benigna.
- Prima o poi tutte le persone con SM manifestano deficit neurologici ben evidenti e persistenti nel tempo.
- La comparsa dei deficit neurologici nella SM non dipende dalla forma della malattia (a ricadute o progressiva).

1. **Quale delle seguenti affermazioni sull’andamento a lungo termine della SM è corretta?**Da studi condotti su persone con SM che non hanno mai fatto la terapia immunomodulante (immunoterapia) risulta che il cammino era pressoché autonomo in ….

- circa 90 persone con SM su 100.
- circa 70 persone con SM su 100.
- circa 50 persone con SM su 100.
- circa 20 persone con SM su 100.

1. **Quale delle seguenti affermazioni sulla scala EDSS scale è corretta?**

- La scala EDSS permette di individuare tutti i deficit neurologici rilevanti della persona con SM.
- I punteggi EDSS tra 4.0 e 7.0 sono determinati principalmente dalla distanza che un paziente è in grado di percorrere camminando.
- Ad un aumento dei deficit neurologici corrisponde sempre un aumento del punteggio EDSS.
- I cambiamenti nelle funzioni visive e cognitive sono ben misurati dalla scala EDSS.

1. **Quale delle seguenti affermazioni è corretta?**

Uno studio randomizzato, controllato con placebo e in doppio cieco, è……

- … una sperimentazione clinica nella quale un nuovo farmaco è confrontato con un vecchio farmaco.
- … una sperimentazione clinica nella quale un farmaco è confrontato con un falso farmaco (placebo). I pazienti vengono assegnati in modo casuale a ricevere il farmaco o il placebo. Sia il medico che il paziente non sanno chi assume il farmaco e chi il placebo.
- … una sperimentazione clinica nella quale un farmaco è confrontato con un falso farmaco (placebo). I pazienti vengono assegnati in modo casuale a ricevere il farmaco o il placebo. I medici che partecipano alla sperimentazione sanno quali dei loro pazienti ricevono il farmaco o il placebo.
- … una sperimentazione clinica nella quale i pazienti, con gli occhi bendati due volte, provano farmaci diversi.

1. **Le tre domande seguenti (8a, 8b, 8c) riguardano la stabilità dei deficit neurologici nelle sperimentazioni cliniche (trial) con gli interferoni nella SM.**

**Per favore, non tralasci nessuna delle domande.**

1. **Quale delle seguenti affermazioni sui risultati nel gruppo trattato con placebo è corretta?**

Stabilità della SM con placebo:

Pensi a 100 persone con SM a ricadute, tutte con 2 ricadute all’anno. Quante di

queste 100 persone possono attendersi che il loro grado di deficit neurologico

rimanga stabile nei successivi due anni di trattamento con placebo?

- ca. 15 su 100.
- ca. 25 su 100.
- ca. 40 su 100.
- ca. 70 su 100.
- ca. 80 su 100.

1. **Quale delle seguenti affermazioni sui risultati nel gruppo trattato con interferone è corretta?**

Stabilità con interferone:
Pensi a 100 persone con a ricadute, tutte con 2 ricadute all’anno. Quante di queste 100 persone possono attendersi che il loro grado di deficit neurologico rimanga stabile nei successivi due anni di trattamento con interferone?

- ca. 15 su 100.
- ca. 25 su 100.
- ca. 40 su 100.
- ca. 70 su 100.
- ca. 80 su 100.

1. **Quale delle seguenti affermazioni sui risultati del trattamento con interferone rispetto al placebo è corretta?**

In altre parole:

Rispondendo alla domanda 8a, ha indicato quante delle 100 persone rimarranno stabili, senza terapia, nei due anni successivi.

Per cui, quante persone in più rimarranno stabili grazie alla terapia con interferone (ovvero, oltre a quelle stabili senza terapia)?

- ca. 10 su 100.
- ca. 25 su 100.
- ca. 45 su 100.
- ca. 55 su 100.
- ca. 65 su 100.

1. **Quale delle seguenti affermazioni sulla terapia per le diverse forme di SM è corretta?**

- Al momento non ci sono sperimentazioni cliniche che provino l’efficacia delle terapie per le persone ai primi segni e/o sintomi di SM.
- Al momento non ci sono sperimentazioni cliniche che provino l’efficacia delle terapie per le persone con SM a ricadute.
- Al momento non ci sono sperimentazioni cliniche che provino l’efficacia delle terapie per le persone con SM progressiva secondaria.
- Al momento non ci sono sperimentazioni cliniche che provino l’efficacia delle terapie per le persone con SM progressiva primaria.

**10. Quale delle seguenti affermazioni sul Copaxone**^®^ **(Glatiramer acetato) è corretta?**

- L’efficacia del Copaxone® nel ridurre la frequenza delle ricadute è paragonabile a quella degli interferoni.
- L’efficacia del Copaxone® nel ridurre la frequenza delle ricadute è superiore a quella degli interferoni.
- L’efficacia del Copaxone® nel ridurre la frequenza delle ricadute è inferiore a quella degli interferoni.
- Non è possibile confrontare l’efficacia del Copaxone® con quella degli interferoni nel ridurre la frequenza delle ricadute.

1. **Quale delle seguenti affermazioni relative ai sintomi “simil-influenzali”, che sono un effetto collaterale dell’interferone, è corretta?**

- Tutte le persone con SM hanno sintomi “simil-influenzali” almeno una volta durante la terapia con interferone.
- I sintomi “simil-influenzali” possono comparire solo all’inizio della terapia con interferone.
- Circa 50 persone con SM su 100 hanno sintomi “simil-influenzali” all’inizio della terapia con interferone.
- Circa 10 persone con SM su 100 hanno sintomi “simil-influenzali” all’inizio della terapia con interferone.

1. **Quale delle seguenti affermazioni sulle terapie orali (in compresse) per la SM è corretta?**

- Il Gilenya^®^ (Fingolimod) è più efficace del Tysabri® (Natalizumab).
- Il Gilenya^®^ (Fingolimod) ha effetti collaterali trascurabili.
- L’efficacia del Tecfidera^®^ (Fumarato) è simile a quella degli interferoni.
- L’Aubagio^®^ (Teriflunomide) è più efficace degli interferoni.

1. **Quale delle seguenti affermazioni sul Tysabri® (Natalizumab) è corretta?**

- Circa 40 su 100 persone con SM a ricadute trattate per due anni con Tysabri®, non presenta un aumento dei deficit neurologici grazie alla terapia.
- Oltre 50 pazienti su 100 trattati con Tysabri® va incontro ad effetti collaterali che insorgono improvvisamente, come conseguenza del trattamento con questa terapia.
- Circa 3 pazienti su 1000 trattati con Tysabri® sviluppa una grave encefalopatia virale, chiamata leucoencefalopatia multifocale progressiva (PML).
- Oltre agli interferoni, il Tysabri® è un trattamento di elezione per la SM progressiva secondaria.

1. **Quale delle seguenti affermazioni su gravidanza e SM è corretta?**

- La frequenza delle ricadute aumenta durante la gravidanza.
- La contraccezione è necessaria per ogni tipo di terapia per la SM.
- Un bambino ogni 50 con un genitore con la SM avrà egli stesso la malattia.
- L’allattamento al seno aumenta il rischio di ricadute.

1. **Quale delle seguenti affermazioni su medicina complementare ed integratori alimentari è corretta?**

- Gli studi hanno dimostrato che la terapia con enzimi riduce la frequenza delle ricadute.
- Gli studi hanno dimostrato che gli acidi poli-insaturi (es. olio di pesce, olio di enotera) rallenta la progressione della malattia.
- Non vi sono dati convincenti relativi agli effetti delle medicine complementari e degli integratori alimentari sull’attività di malattia.
- Gli studi hanno dimostrato che la vitamina D riduce la frequenza delle ricadute.

**Ora per le seguenti domande indichi la risposta che ritiene sbagliata.**

1. **Quale delle seguenti affermazioni relative alla diagnosi è sbagliata?**

- La diagnosi di SM può essere confermata se i sintomi tipici della malattia e le caratteristiche alterazioni alla RMN si presentano contemporaneamente, e se una successiva RMN eseguita poco dopo la prima evidenzia nuove aree di infiammazione.
- Nella maggior parte dei casi, la SM può essere diagnosticata solo dopo un po’ che la malattia abbia avuto il suo corso.
- Qualche volta può essere difficile diagnosticare la SM con assoluta certezza.
- La SM può essere diagnosticata solo in presenza di anticorpi nel liquido cerebro-spinale, che sono riscontrabili soltanto nella SM.

1. **Quale delle seguenti affermazioni sulle ricadute è sbagliata?**

- Le ricadute sono sintomi nuovi che compaiono nel giro di alcuni giorni o settimane.
- Le ricadute sono sintomi vecchi che si accentuano solo per poche ore.
- Le ricadute sono sintomi vecchi che si intensificano, o nuovi sintomi che durano almeno 24 ore.
- A volte può essere difficile distinguere le ricadute dalle fluttuazioni giornaliere dei sintomi della SM.

1. **Quale delle seguenti affermazioni sulle terapie per la SM è sbagliata?**

- Le terapie per la SM servono a guarire dalla malattia.
- Le terapie per la SM funzionano al meglio nelle forme di SM a ricadute.
- Le terapie per la SM possono rallentare la progressione della malattia.
- Le terapie per la SM possono ridurre la frequenza delle ricadute.

1. **Quale delle seguenti affermazioni sui farmaci per l’immunoterapia è sbagliata?**

- Il Mitoxantrone è stato autorizzato per il trattamento della SM a ricadute.
- Alcuni tipi di interferone sono stati autorizzati per il trattamento della SM progressiva secondaria.
- Il Tysabri® (Natalizumab) è stato autorizzato per il trattamento della SM a ricadute.
- Il Gilenya^®^ (Fingolimod) è stato autorizzato per il trattamento della SM progressiva.

**Molte grazie per il suo contributo!**

Ovaj upitnik treba da proceni vaše poznavanje multiple skleroze. On je posebno napravljen da proceni poznavanje rizika u obrazovnim programima za bolesnike koji razmatraju primenu imunoterapije. Možda ćete imati poteškoće pri odgovaranju na pitanja. Ali, imajte u vidu: ovo ne predstavlja test da proceni da li ste ''dobri'' ili ''loši''.

On je napravljen kao pokazatelj zdravstvenim profesionalcima kako da procene potrebu za davanjem podrške kada vas informišu o bolesti.

Molimo Vas da pročitate svako pitanje i obeležite samo jedan odgovor.

Molimo vas da pročitate sledeća pitanja i obeležite jedan odgovor u svakoj grupi koji smatrate tačnim.

**1. Koja je od sledećih izjava o relapsima tačna?**

- Česti relapsi ukazuju na brže pogoršanje stepena oštećenja u budućnosti, kad god da se pojavljuju tokom MS.
- Česti relapsi tokom prve 2 godine bolesti ukazuju na brže pogoršanje stepena oštećenja u budućnosti.
- Manji broj relapsa tokom bolesti je znak poboljšanja.
- Teški relapsi u bilo kojoj fazi bolesti ukazuju na brža pogoršanja stepena oštećenja u budućnosti.

**2.**  **Koja je od sledećih izjava o magnetnoj rezonanci (MRI) tačna?**

- Stepen oštećenja kod pacijenata može biti utvrđen magnetnom rezonancom.
- Prebojavanje kontrastom (gadolinijumom) vidljivo na magnetnoj rezonanci znak je postojećeg zapaljenja.
- Svi pacijenti koji boluju od MS treba da urade magnetnu rezonancu bar jednom godišnje.
- Zone zapaljenja koje se vide na magnetnoj rezonanci (bele tačke) ukazuju na propadanje nervnih omotača i nervnih ćelija.

**3. Koja је od sledećih izjava o tipovima МS tačna?**

- Kada se postavi dijagnoza MS, istovremeno može i da se odredi tok bolesti.
- Uopšte uzevši, prelazak iz relapsne u hronično progresivnu formu bolesti može da se odredi samo retrospektivno.
- Vrlo mali broj MS bolesnika ima od početka relapsnu formu bolesti.
- Tok bolesti nije od značaja za donošenje odluke o terapiji.

**4. Koja је od sledećih izjava o tipovima МS tačna?**

- Do 30 od 100 MS pacijenata će ostati bez značajnog stepena oštećenja, čak i posle 20 godina trajanja MS (benigna MS).
- Ne postoji benigni oblik MS.
- Pre ili kasnije kod svih pacijenata koji boluju od MS razviju se jasno uočljiva, trajna oštećenja.
- Razvoj oštećenja kod MS ne zavisi od tipa bolesti (relapsne ili progresivne MS).

**5. Koja је od sledećih izjava o dugoročnom toku MS tačna?**

Studije koje su se bavile pacijentima koji boluju od MS i koji nikada nisu primali

imunoterapiju, pokazuju da je posle 15 godina mogućnost hoda bila skoro neograničena

kod....

- oko 90 od 100 bolesnika.
- oko 70 od 100 bolesnika.
- oko 50 od 100 bolesnika.
- oko 20 od 100 bolesnika.

**6. Koja је od sledećih izjava o EDSS skali tačna?**

- Na osnovu EDSS skale može adekvatno da se odredi stepen oštećenja kod bolesnika.
- EDSS skorovi od 4 do 7 su pretežno određeni rastojanjem koje pacijenti mogu da pređu.
- Povećanja stepena oštećenja se uvek ogledaju i povećanjem na EDSS skali.
- Poremećaji vida i kognitivnih sposobnosti se mogu dobro proceniti pomoću EDSS skali.

**7. Koja је od sledećih izjava tačna?**

Duplo slepa, randomizovana placebo kontrolisana studija je...

- studija u kojoj se ispituje novi lek u poređenju sa starim.
- studija u kojoj se ispituje novi lek u poređenju s placebom. Pacijenti metodom slučajnog izbora dobijaju lek ili placebo. Ni lekar ni pacijent ne znaju ko dobija koju supstancu.
- studija u kojoj se ispituje novi lek u poređenju s placebom. Pacijenti metodom slučajnog izbora dobijaju lek ili placebo. Lekari koji vrše ispitivanje upoznati su sa time koja se supstanca daje njihovim pacijentima.
- studija u kojoj pacijenti sa dvostrukim povezom preko očiju primaju različite lekove.

**8. Sledeća tri pitanja se odnose na stepen oštećenja u studijama sa interferonom u**

**MS (8a, 8b, 8c). Molimo vas da odgovorite na sva tri pitanja.**

1. **Koja je od sledećih izjava o rezultatima terapije placebom tačna?**

Stabilnost MS kada se uzima placebo:
Zamislite 100 pacijenata sa relapsnom MS koji svi imaju 2 relapsa godišnje. Koliko od ovih 100 pacijenata može da očekuje da njihov stepen oštećenja ostane stabilan na terapiji placebom sledeće 2 godine?

- oko 15 od 100
- oko 25 od 100
- oko 40 od 100
- oko 70 od 100
- oko 80 od 100

1. **Koja je od sledećih izjava o rezultatima terapije interferonom tačna:**

Stabilnost MS kada se prima interferon:
 Zamislite 100 pacijenata sa relapsnom MS koji svi imaju 2 relapsa godišnje. Koliko od

ovih 100 pacijenata može očekivati da njihov stepen oštećenja ostane stabilan na

terapiji interferonom sledeće 2 godine?

- oko 15 od 100
- oko 25 od 100
- oko 40 od 100
- oko 70 od 100
- oko 80 od 100

1. **Koja je od sledećih izjava o terapiji interferonom u odnosu na placebo tačna?**

Drugačije rečeno U odgovoru na pitanje 8a rekli ste koliko bi od 100 pacijenata ostalo stabilno bez terapije u naredne 2 godine. Sada, koliko bi još pacijenata ostalo stabilno zahvaljujući terapiji interferonom (tj. dodatno u odnosu na one koji bi ostali stabilni bez terapije)?

- oko 10 od 100
- oko 25 od 100
- oko 45 od 100
- oko 55 od 100
- oko 65 od 100

**9. Koja је od sledećih izjava o lečenju različitih tipova MS tačna?**

- Trenutno ne postoje studije koje dokazuju delotvornost lečenja pacijenata kod kojih su se javili prvi znaci i simptomi MS.
- Trenutno ne postoje studije koje dokazuju delotvornost lečenja pacijenata koji imaju relapsnu MS.
- Trenutno ne postoje studije koje koje dokazuju delotvornost lečenja pacijenata koji imaju sekundarno progresivnu MS.
- Trenutno ne postoje studije koje koje dokazuju delotvornost lečenja pacijenata koji imaju primarno progresivnu MS.

**10. Koja је od sledećih izjava o Copaxonu® (Glatiramer acetatu) tačna?**

- Delotvornost Copaxona® u redukciji učestalosti relapsa je slična interferonima.
- Delotvornost Copaxona® u smanjenju učestalosti relapsa je bolja nego kod interferona.
- Delotvornost Copaxona® u smanjenju učestalosti relapsa je lošija nego kod interferona.
- Nije moguće uporediti delotvornost Kopaksona Copaxona® i interferona u smanjenju učestalosti relapsa.

**11. Koja је od sledećih izjava o simptomima sličnim gripu, neželjenom efektu**

**interferona, tačna?**

- Svi pacijenti koji boluju od MS iskusiće simptome slične gripu najmanje jednom u nekoj fazi terapije interferonom.
- Simptomi slični gripu se javljaju samo na početku terapije interferonom.
- Oko 50 od svakih 100 MS bolesnika će imati simptome slične gripu na početku terapije interferonom.
- Oko 10 od svakih 100 MS bolesnika će imati simptome slične gripu na početku terapije interferonom.

**12. Koja је od sledećih izjava o terapijama MS (sa tabletama) tačna?**

- Gilenya® (Fingolimod) je efikasnija od Tysabri-ja.
- Gilenya® (Fingolimod) skoro da nema neželjene efekte.
- Efikasnost Tecfidere® (Fumarat) je slična onoj koju ima interferon.
- Aubagio® (Teriflunomide) je efikasniji od interferona.

**13. Koja је od sledećih izjava o Tysabriju® (Natalizumabu) tačna?**

- Ukoliko lečenje Tysabrijem® traje 2 godine, oko 40 od svakih 100 pacijenata sa relapsnom MS neće imati progresiju stepena oštećenja zahvaljujući leku.
- Kod više od 50 od svalih 100 pacijenata iznenada se pojavljuju neželjena dejstva kao rezultat terapije Tisabrijem®.
- Oko 3 od 1000 pacijenata lečenih Tisabrijem® ima ozbiljnu virusnu infekciju mozga (Progresivna Multifokalna Leukoencefalopatija/PML).
- Pored interferona, Tysabri® je lek prvog izbora za sekundarno progresivnu MS.

**14. Koja је od sledećih tvrdnji koja se odnosi na trudnoću I MS tačna?**

- Učestalost relapsa se povećava tokom trudnoće.
- Kontracepcija je obavezna tokom tretmana MS.
- Jedno od 50-oro dece čiji jedan roditelj ima MS oboleće od ove bolesti.
- Dojenje povećava rizik od relapsa.

**15. Koja је od sledećih tvrdnji koje se odnose na alternativnu medicine i nutritivne**

**suplemente tačna?**

- Studije su pokazale da enzimska terapija smanjuje učestalost relapse.
- Studije su pokazale da nezasićene masne kiseline (npr. riblje ulje, ulje jagorčevine) usporavaju progresiju bolesti.
- Ne postoji studija koja ubedljivo pokazuje da alternativna medicina ili nutritivni suplementi utiču na aktivnost bolesti u MS.
- Studije su pokazale da vitamin D smanjuje učestalost relapsa.

**Zdaj, za naslednje izjave, označite en odgovor, za katerega menite, da je napačen.**

**16. Koja je od sledećih izjava o dijagnozi pogrešna?**

- Dijagnoza MS se može potvrditi ukoliko se istovremeno uoče tipični simptomi i karakteristični MRI nalazi i ako naredni MRI, načinjen ubrzo nakon prvog, potvrdi postojanje novih zapaljenjskih područja.
- U većini slučajeva MS se može dijagnostikovati samo ako bolest traje neko vreme.
- Nekada može da bude teško da se dijagnoza MS postavi bez ikakve sumnje u to.
- MS se može dijagnostikovati samo na osnovu antitela iz likvora, a koja postoje samo kod ove bolesti.

**17. Koja je od sledećih izjava o relapsima pogrešna?**

- Relapsi su novi simptomi koji se razvijaju tokom nekoliko dana ili nedelja.
- Relapsi su stari simptomi koji buknu na samo par sati.
- Relapsi su intenzivirani stari ili novi simptomi koji traju najmanje 24 časa.
- Nekada je teško razlikovati relapse od svakodnevnih variranja simptoma MS.

**18. Koja je od sledećih izjava o terapijama MS pogrešna?**

- Cilj terapija MS je izlečenje bolesti.
- Terapije MS daju najbolje rezultate u slučajevima relapsne MS.
- Terapije MS mogu da uspore napredovanje bolesti.
- Terapije MS mogu da smanje učestalost relapsa.

**19. Koja je od sledećih izjava o lekovima za imunoterapiju pogrešna?**

- Mitoksantron je odobren za lečenje relapsne MS.
- Neki interferoni su odobreni za lečenje sekundarno progresivne MS.
- Tysabri® (Natalizumab) je odobren za lečenje relapsne MS.
- Gilenya® (Fingolimod) je odobrena za tretman hronične MS.

**Hvala za vašo pomoč!**

Este cuestionario evalúa su conocimiento acerca de la EM. Se ha diseñado especialmente para valorar cuál es el grado de conocimiento incluido en los materiales educativos dirigidos a pacientes que están el proceso de tomar una decisión acerca de la medicación para la EM. Aun cuando pueda considerar difícil contestar a alguna de las preguntas, tenga en cuenta que no se trata de juzgar si su conocimiento es »bueno« o«malo«.

Este cuestionario se diseñó como una herramienta para los profesionales sanitarios que ayudara a determinar la necesidad de ofrecer apoyo al informar a los pacientes sobre la enfermedad.

Por favor, lea cada pregunta para marcar una única opción entre las posibles respuestas.

Por favor, lea las siguientes preguntas y marque solamente una respuesta entre las propuestas que usted considere que es la correcta.

**1. ¿Cuál de las siguientes afirmaciones acerca del brote de EM es correcta?**

- Los brotes frecuentes indican un empeoramiento rápido de la discapacidad en un futuro, independientemente de cuando se produzcan durante el curso de la enfermedad.
- Los brotes frecuentes durante los dos primeros años de la enfermedad indican un empeoramiento rápido de la discapacidad en un future.
- Si se producen pocos brotes de la enfermedad, esto es una señal de que se está produciendo una mejora de la enfermedad.
- Brotes graves en cualquier fase del curso de la enfermedad predicen un empeoramiento rápido de la discapacidad en el futuro.

**2. ¿Cuál de las siguientes afirmaciones sobre la resonancia magnética (RM) es**

**correcta?**

- La discapacidad del paciente se puede determinar mediante RM.
- El realce por contraste (gadollinio) que se observa en la RM es un signo de inflamación activa.
- Se debería realizar una RM a cada paciente de EM al menos una vez al año.
- Las áreas de inflamación observadas en la RM (puntos blancos) indican la destrucción de la vaina que recubre los axones de las células nerviosas y de las propias células nerviosas.

**3. ¿Cuál de las siguientes afirmaciones sobre los tipos de EM es correcta??**

- Cuando se realiza un diagnóstico de EM también se puede determinar el curso de la enfermedad.
- En general, el paso de una fase recurrente a una progresiva crónica sólo se puede determinar retrospectivamente.
- Muy pocos pacientes de EM presentan un curso recurrente desde el inicio.
- No es necesario tener en cuenta el curso de la enfermedad para tomar una decisión sobre medicación para la EM.

**4. ¿Cuál de las siguientes afirmaciones sobre los tipos de EM es correcta??**

- Hasta 30 de cada 100 pacientes con EM no experimentará discapacidad relevante 20 años o más desde el diagnóstico (EM benigna).
- La EM benigna no existe.
- Tarde o temprano todos los pacientes con EM acaban desarrollando déficits visibles y de larga duración.
- El desarrollo de déficits en EM no depende del tipo de enfermedad (recurrente o progresivo).

**5. ¿Cuál de las siguientes afirmaciones sobre la evolución a largo plazo de la EM es**

**correcta?**

Estudios publicados en personas con EM que nunca han tomado inmunoterapia para la

enfermedad muestran que a los 15 años su capacidad para caminar casi sin limitaciones

se mantenía en…

- alrededor de 90 de cada 100 pacientes.
- alrededor de 70 de cada 100 pacientes.
- alrededor de 50 de cada 100 pacientes.
- alrededor de 20 de cada 100 pacientes.

**6. ¿Cuál de las siguientes afirmaciones sobre la escala EDSS es correcta?**

- A partir de la puntuación en la escala EDSS se pueden identificar todos los déficits de relevancia que presenta un paciente.
- Las puntuaciones de EDSS de 4.0 a 7.0 vienen principalmente determinadas por las distancias que un paciente puede caminar.
- Los aumentos en la discapacidad se reflejan siempre en un aumento en la EDSS.
- Los cambios en la visión y el estado cognitivo se miden bien con la escala EDSS.

**7. ¿Cuál de las siguientes afirmaciones es correcta?**

Un ensayo doble-ciego aleatorizado y controlado con placebo es un ensayo...

- en el que se pruebo un nuevo fármaco frente a otro antiguo.
- en el que un fármaco que se prueba frente a un fármaco inerte (placebo). Los pacientes se asignan aleatoriamente para tomar el fármaco o el placebo. Ni el médico ni el paciente saben quien toma qué sustancia.
- en el que un fármaco se prueba frente a placebo. Los pacientes se asignan aleatoriamente para tomar el fármaco o el placebo. El médico conoce qué sustancia toma cada paciente.
- en el que los paciente prueban un fármaco con los ojos vendados dos veces.

**8. Las tres preguntas que siguen (8a, 8b, 8c) se refieren a la estabilidad de la**

**discapacidad observada en ensayos con interferones. Por favor, responda a las**

**tres preguntas.**

1. **¿Cuál de las siguientes afirmaciones sobre los resultados del tratamiento con placebo es correcta?**

Estabilidad de la EM tomado placebo:

imagine 100 pacientes con EM en brotes los cuales todos han tenido dos brotes anuales. ¿Cuántos de estos 100 pacientes pueden esperar que su grado de discapacidad permanezca estable en los próximos 2 años siguiendo con tratamiento con placebo?

- alrededor de 15 de los 100
- alrededor de 25 de los 100
- alrededor de 40 de los 100
- alrededor de 70 de los 100
- alrededor de 80 de los 100

1. **¿Cuál de las siguientes afirmaciones sobre los resultados del tratamiento con interferón es correcta?**Estabilidad de la EM tomado interferón beta:

imagine 100 pacientes con EM en brotes los cuales todos han tenido dos brotes anuales. ¿Cuántos de estos 100 pacientes pueden esperar que su grado de discapacidad permanezca estable en los próximos 2 años siguiendo con tratamiento con interferon?

- alrededor de 15 de los 100
- alrededor de 25 de los 100
- alrededor de 40 de los 100
- alrededor de 70 de los 100
- alrededor de 80 de los 100

1. **¿Cuál de las siguientes afirmaciones sobre el tratamiento con interferón frente a placebo es correcta?**En la pregunta 8a respondió usted acerca de cuántos de los 100 pacientes permanecerían estables sin terapia en los siguientes 2 años. Bajo el supuesto del enunciado de la pregunta actual (8c), ¿cuántos **más** pacientes permanecerán estables como resultado del tratamiento con interferón (es decir, **añadidos** a los que ya nos indicó para los que no tomen tratamiento)?

- alrededor de 10 de los 100
- alrededor de 25 de los 100
- alrededor de 45 de los 100
- alrededor de 55 de los 100
- alrededor de 65 de los 100

**9. ¿Cuál de las siguientes afirmaciones sobre el tratamiento de los distintos tipos de**

**EM es correcta?**

- Actualmente no existe ningún ensayo que demuestre la eficacia de fármacos destinados al tratamiento de pacientes con primeros signos y síntomas de EM.
- Actualmente no existe ningún ensayo que demuestre la eficacia de fármacos destinados al tratamiento de pacientes con formas recurrentes de EM.
- Actualmente no existe ningún ensayo que demuestre la eficacia de de fármacos destinados al tratamiento de pacientes con EM secundariamente progresiva.
- Actualmente no existe ningún ensayo que demuestre la eficacia de de fármacos destinados al tratamiento de pacientes con EM primariamente progresiva.

**10. ¿Cuál de las siguientes afirmaciones relativas a Copaxone® (acetato de**

**glatirámero) es correcta?**

- La efectividad de Copaxone® en la reducción de la tasa de brotes es comparable a la de los interferones.
- La efectividad de Copaxone® en la reducción de la tasa de brotes es superior a la de los interferones.
- La efectividad de Copaxone® en la reducción de la tasa de brotes es inferior a la de los interferones.
- No es posible comparar la efectividad de Copaxone® y la de los interferones en la reducción de la tasa de brotes.

**11. ¿Cuál de la siguientes afirmaciones sobre el síndrome pseudogripal, un efecto**

**secundario del tratamiento con interferón, es correcta?**

- Todos los pacientes con EM padecerán síntomas pseudogripales por lo menos una vez a lo largo del tratamiento con interferón.
- El síndrome pseudogripal aparece solamente al inicio del tratamiento con interferón.
- Alrededor de 50 de cada 100 pacientes con EM padecen un síndrome pseudogripal al inicio del tratamiento con interferón.
- Alrededor de 10 de cada 100 pacientes con EM padecen un síndrome pseudogripal al inicio del tratamiento con interferón.

**12. ¿Cuál de las siguientes afirmaciones sobre la terapia oral con pastillas para la EM**

**es correcta?**

- Gilenya® (fingolimod) es más eficaz que Tysabri® (natalizumab).
- Gilenya® (fingolimod) no tiene prácticamente efectos secundarios.
- Tecfidera® (dimetil fumarato) presenta una eficacia similar a los interferones.
- Aubagio® (teriflunomida) es más efectiva que los interferones.

**13. ¿Cuál de las siguientes afirmaciones sobre Tysabri® (natalizumab) es correcta?**

- Como resultado del fármaco, al cumplir 2 años de tratamiento con Tysabri® alrededor de 40 de cada 100 pacientes con EM recurrente no ha experimentado incremento en discapacidad.
- Más de 50 de cada 100 pacientes experimentan efectos secundarios de presentación repentina como resultado del tratamiento con Tysabri®.
- Alrededor de 3 de cada 1000 pacientes tratados con Tysabri® sufre un infección vírica cerebral aguda (Leucoencefalopatía Multifocal Progresiva – LMP).
- Además de los interferones, Tysabri® es tratamiento de primera línea para el tratamiento de EM secundariamente progresiva.

**14. ¿Cuál de las siguientes afirmaciones relativas al embarazo y la EM es correcta?**

- La tasa de brotes se incrementa durante el embarazo.
- La contracepción és obligatoria durante el tratamiento de la EM.
- Uno de cada 50 niños con un progenitor afecto/a de EM. también tendrá la enfermedad.
- La lactancia incrementa el riesgo de sufrir un brote.

**15. ¿Cuál de las siguientes afirmaciones sobre la medicina alternativa y los**

**suplementos nutricionales es correcta?**

- Existen estudios que prueban que el tratamiento con encimas reduce la tasa de brotes.
- Existen estudios que prueban que los ácidos poli-insaturados (como p. ej. los aceites de pescado, aceite de onagra, etc) frenan la progresión de la enfermedad.
- No existen estudios convincentes que prueben que la medicina alternativa o los suplementos nutricionales ejerzan influencia alguna sobre la actividad de la enfermedad en EM.
- Existen estudios que prueban que la vitamina D reduce la tasa de brotes.

**Ahora, para las siguientes afirmaciones, marque la respuesta que considere errónea.**

**16. ¿Cuál de las siguientes afirmaciones sobre el diagnóstico es errónea?**

- Se puede confirmar el diagnóstico de EM si se observan síntomas característicos y hallazgos en la RM simultáneamente y una RM posterior realizada al poco tiempo muestra evidencia de nuevas áreas de inflamación.
- En la mayoría de casos, la EM no se puede diagnosticar hasta pasado un tiempo de la manifestación de enfermedad.
- A veces puede resultar difícil diagnosticar la EM más allá de toda duda.
- La EM se puede diagnosticar solamente si se encuentran anticuerpos en el líquido céfalorraquideo que sólo se hallan en la EM.

**17. ¿Cuál de las siguientes afirmaciones sobre los brotes es errónea?**

- Un brote es un conjunto de nuevos síntomas que se desarrollan a lo largo de unos días o semanas.
- Un brote representa síntomas antiguos que afloran tan solo por unas horas.
- Un brote representa síntomas antiguos o nuevos que duran más de 24 horas.
- A veces, puede resultar difícil distinguir entre un brote y fluctuaciones propias de los síntomas de EM.

**18. ¿Cuál de las siguientes afirmaciones sobre el tratamiento de la EM es errónea?**

- El tratamiento de la EM aspira a curar la enfermedad.
- El tratamiento de la EM funciona mejor en los casos de EM remitente-recurrente.
- El tratamiento de la EM puede retrasar la progresión de la enfermedad.
- El tratamiento de la EM puede reducir la frecuencia de los brotes.

**19. ¿Cuál de las siguientes afirmaciones sobre fármacos inmunoterapéuticos es**

**errónea?**

- La mitoxantrona está aprobada para el tratamiento de la EM remitente-recurrente.
- Algunos de los interferones están aprobados para el tratamiento de la EM secundariamente-progresiva.
- El natalizumab (Tysabri®) está aprobado para el tratamiento de la EM en brotes.
- El fingolimod (Gilenya®) está aprobado para el tratamiento de la EM crónica.

**Muchas gracias por tu ayuda!**

Deze vragenlijst onderzoekt uw kennis over MS. De vragenlijst is speciaal ontwikkeld om te onderzoeken of mensen met MS die starten met ziekteremmende medicatie voldoende kennis hebben van de risico's van deze behandeling. Het kan zijn dat u de vragen moeilijk te beantwoorden vindt. Houd daarbij in gedachten dat uw antwoorden niet als "goed" of "slecht" beoordeeld worden.

De vragenlijst is namelijk ontwikkeld als een meetinstrument voor gezondheidswerkers om de behoefte aan ondersteuning in kaart te brengen wanneer zij iemand met MS informeren over het ziektebeeld.

Lees elke vraag aandachtig en vink steeds het antwoord dat van toepassing is aan.

Lees de volgende vragen en vink het antwoord in elke groep aan dat u het juiste vindt.

**1**. **Welke van de volgende uitspraken over exacerbaties (terugvallen) is juist? ***

Kies a.u.b. een van de volgende mogelijkheden:

- Frequente exacerbaties wijzen op een toename van beperkingen in de toekomst, ongeacht wanneer ze optreden in het verloop van de MS.
- Frequente exacerbaties gedurende de eerste 2 jaar na de diagnose MS wijzen op een snellere toename van beperkingen in de toekomst.
- Indien er minder exacerbaties tijdens de ziekte optreden, is dat een teken van een verbetering van de ziekte.
- Ernstige exacerbaties tijdens het ziektebeloop wijzen op een snellere toename van beperkingen in de toekomst.

**2.** **Welke van de volgende uitspraken over MRI is juist?***

Kies a.u.b. een van de volgende mogelijkheden

- De beperkingen van iemand met MS kunnen worden bepaald op basis van de MRI beelden.
- Zichtbare aankleuring door contrastmiddel (gadolinium) op de MRI is een teken van actieve ontsteking.
- Alle mensen met MS moeten minstens één keer per jaar een MRI ondergaan.
- Ontstekingshaarden op de MRI scan (witte vlekken) wijzen op kapot gaan van myeline en zenuwwcellen.

**3. Welke van de volgende uitspraken over de beloopsvormen van MS is juist?***

Kies a.u.b. een van de volgende mogelijkheden

- Wanneer de diagnose MS wordt gesteld kan ook de beloopsvorm bepaald worden.
- In het algemeen kan de overgang van Relapsing Remitting MS naar Secundair Progressieve MS alleen maar achteraf bepaald worden.
- Zeer weinig mensen met MS hebben een Relapsing Remitting beloopsvorm van MS wanneer zij de diagnose krijgen.
- De beloopsvorm van MS is niet relevant voor de keuze om wel of niet te behandelen.

**4. Welke van de volgende uitspraken over de beloopsvormen van MS is juist?***

Kies a.u.b. een van de volgende mogelijkheden

- Tot 30% van alle mensen met MS heeft een milde (benigne) beloopsvorm, zelfs 20 jaar na de diagnose.
- Milde MS bestaat niet.
- Vroeg of laat ontwikkelen alle mensen met MS duidelijk merkbare, blijvende beperkingen.
- Het ontwikkelen van beperkingen door de MS is niet afhankelijk van de beloopsvorm van de MS (Relapsing of progressieve MS).

**5. Welke van de volgende uitspraken over het beloop van MS op de lange**

**termijn is juist?**

Studies onder mensen met MS die nooit een ziekteremmende behandeling

ondergingen tonen aan dat na 15 jaar het vermogen tot lopen bijna onbeperkt was

bij... *

Kies a.u.b. een van de volgende mogelijkheden

- Ongeveer 90% van alle mensen met MS
- Ongeveer 70% van alle mensen met MS
- Ongeveer 50% van alle mensen met MS
- Ongeveer 20% van alle mensen met MS

**6. Welke van de volgende uitspraken over de EDSS-schaal is juist? ***

Kies a.u.b. een van de volgende mogelijkheden

- Met behulp van de EDSS schaal kan men alle relevante beperkingen van iemand met MS in kaart brengen.
- Een EDSS-score tussen 4,0 en 7,0 wordt voornamelijk bepaald door de afstand die de persoon met MS kan lopen.
- Een toename van beperkingen komt altijd tot uitdrukking in een toename van de EDSS-score.
- Veranderingen in het gezichtsvermogen en cognitieve vaardigheden worden met de EDSS-schaal goed in kaart gebracht.

**7. Welke van de volgende beweringen is juist?** **Een dubbelblinde, gerandomiseerde, placebo-gecontroleerde studie is ... ***

Kies a.u.b. een van de volgende mogelijkheden:

- Een studie waarbij de werking van een nieuw medicijn is vergeleken met de werking van een bestaand medicijn.
- Een studie waarin de werking van een medicijn wordt vergeleken met de werking van een nep medicijn (placebo). De deelnemers aan de studie worden willekeurig toegewezen aan het middel of het placebo. Noch arts noch patiënt weet wie welk middel krijgt.
- Een proef waarin de werking van een medicijn wordt vergeleken ten opzichte van een placebo. De deelnemers aan de studie worden willekeurig toegewezen aan het middel of het placebo. De studie-artsen worden geïnformeerd over welke stof wordt gegeven aan hun patiënten.
- Een proef waarin deelnemers met geblinddoekte ogen twee keer een verschillende medicijn uitproberen.

**8. De volgende drie vragen gaan over het stabiel blijven van de MS in de**

**studies naar het effect van Interferonen op MS.**

1. **Welke van de volgende uitspraken over de resultaten van een behandeling met een placebo is juist?**Stabiel blijven van MS bij het behandelen met een placebo:

Stel je 100 mensen met Relapsing Remitting MS voor die allemaal 2 exacerbaties per jaar doormaken. Hoeveel van deze 100 mensen met MS kunnen verwachten dat hun niveau van beperkingen binnen de komende 2 jaar stabiel blijft op de behandeling met een placebo?*

Kies a.u.b. een van de volgende mogelijkheden:

- Ongeveer 15%
- Ongeveer 25%
- Ongeveer 40%
- Ongeveer 70%
- Ongeveer 80%

1. **Welke van de volgende uitspraken over de resultaten van een behandeling met Interferon is juist?**Stability Het stabiel blijven van de MS bij het behandelen met Interferon:

Stel je 100 mensen met Relapsing Remitting MS voor die allemaal 2 exacerbaties per jaar doormaken. Hoeveel van deze 100 mensen met MS kunnen verwachten dat hun niveau van beperkingen de komende 2 jaar stabiel blijft op de behandeling met interferon?*

Kies a.u.b. een van de volgende mogelijkheden:

- Ongeveer 15%
- Ongeveer 25%
- Ongeveer 40%
- Ongeveer 70%
- Ongeveer 80%

1. **Welke van de volgende uitspraken over de behandeling met Interferon versus placebo is juist?**

Met andere woorden:

In het antwoord op vraag over een placebo gaf u aan hoeveel procent van de mensen met MS de komende 2 jaar stabiel zouden blijven zonder behandeling. Hoeveel mensen met MS zouden stabiel blijven ten gevolge van de behandeling met Interferon (dus bovenopdegenen die zonder behandeling stabiel blijft)? *

Kies a.u.b. een van de volgende mogelijkheden:

- Ongeveer 10%
- Ongeveer 25%
- Ongeveer 45%
- Ongeveer 55%
- Ongeveer 65%

**9. Welke van de volgende uitspraken over de behandeling van de**

**verschillende beloopsvormen van MS is juist? ***

Kies a.u.b. een van de volgende mogelijkheden:

- Er zijn momenteel geen studies waaruit effectiviteit blijkt van de behandeling voor mensen met MS die hun eerste verschijnselen of symptomen ervaren.
- Er zijn geen studies waaruit de effectiviteit blijkt van behandeling voor mensen met Relapsing Remitting MS.
- Er zijn geen studies waaruit de effectiviteit blijkt van de behandeling voor mensen met Secundaire Progressieve MS.
- Er zijn geen studies waaruit de effectiviteit blijkt van de behandeling voor patiënten met Primair Progressieve MS.

**10. Welke van de volgende verklaringen over Copaxone® (glatirameer acetaat)**

**is juist? ***

Kies a.u.b. een van de volgende mogelijkheden:

- De effectiviteit van Copaxone® op het verminderen van het aantal relapses is vergelijkbaar met die van Interferonen.
- De effectiviteit van Copaxone® op het verminderen van het aantal relapses is beter dan die van Interferonen.
- De effectiviteit van Copaxone® op het verminderen van het aantal relapses is slechter dan die van Interferonen.
- Het is niet mogelijk om de effectiviteit op het verminderen van het aantal relapses te vergelijken tussen Copaxone® en Interferonen.

**11. Welke van de volgende uitspraken over griepachtige verschijnselen, een**

**bijwerking van Interferonen, is juist? ***

Kies a.u.b. een van de volgende mogelijkheden:

- Alle mensen met MS ervaren minimaal één keer tijdens de behandeling met Interferon griepachtige verschijnselen.
- Griepachtige verschijnselen worden alleen in het begin van een Interferon behandeling ervaren.
- Ongeveer 50% van de mensen met MS die starten met Interferon ervaren gedurende de eerste periode van de behandeling griepachtige verschijnselen.
- Ongeveer 10% van de mensen met MS die starten met Interferon ervaren gedurende de eerste periode van de behandeling griepachtige verschijnselen.

**12. Welke van de volgende uitspraken over MS-therapieën met tabletten is**

**juist? ***

Kies a.u.b. een van de volgende mogelijkheden:

- Gilenya® (fingolimod) is effectiever dan Tysabri®.
- Gilenya® (fingolimod) heeft nauwelijks bijwerkingen.
- De werkzaamheid van Tecfidera® (Dimethyl fumaraat) is gelijk aan die van de interferonen.
- Aubagio® (Teriflunomide) is effectiever dan Interferonen.

**13. Welke van de volgende uitspraken over Tysabri® (Natalizumab) is juist? ***

Kies a.u.b. een van de volgende mogelijkheden:

- Na 2 jaar behandeling met Tysabri® heeft ongeveer 40% van de mensen met Relapsing Remitting MS geen toename van de beperkingen door MS als gevolg van de behandeling met dit medicijn.
- Meer dan 50% van de mensen met MS ervaren bijwerkingen die plotseling kunnen optreden als gevolg van een behandeling met Tysabri®.
- Ongeveer 3 op de 1000 mensen met MS die worden behandeld met Tysabri® lijden aan een ernstige virale infectie van de hersenen Progressieve Multifocale Leuko-encefalopathie / PML).
- In aanvulling op interferonen is Tysabri® een eerste-keuze behandeling voor Secundaire Progressieve MS.

**14**. **Welke van de volgende stellingen met betrekking tot zwangerschap en MS**

**zijn juist? ***

Kies a.u.b. een van de volgende mogelijkheden:

- Het aantal exacerbaties neemt tijdens de zwangerschap toe.
- Anticonceptiemiddelen zijn verplicht gedurende iedere MS behandeling.
- Eén op de 50 kinderen met een ouder met MS zal zelf ook MS krijgen.
- Borstvoeding verhoogt het risico op een exacerbatie.

**15**. **Welke van de volgende uitspraken voor alternatieve behandelwijzen en**

**voedingssupplementen is juist? ***

Kies a.u.b. een van de volgende mogelijkheden:

- Studies hebben aangetoond dat enzymtherapie het aantal relapses doet afnemen.
- Studies hebben aangetoond dat meervoudig onverzadigde vetzuren (zoals visolie, teunisbloemolie) de ziekteprogressie bij MS vertragen.
- Er zijn geen overtuigende studies waaruit blijkt dat alternatieve behandelwijzen of voedingssupplementen invloed hebben op de ziekteactiviteit bij MS.
- Studies hebben aangetoond dat vitamine D het aantal exacerbaties doet afnemen.

**Nu, voor de volgende verklaringen, vink alstublieft het ene antwoord aan dat u als beschouwt niet juist? ***

**16. Welke van de volgende uitspraken met betrekking tot de diagnose is niet**

**juist? ***

Kies a.u.b. een van de volgende mogelijkheden:

- De diagnose MS kan worden bevestigd als typische symptomen en specifieke MRI bevindingen gelijktijdig voorkomen en een volgende MRI die korte tijd later gemaakt wordt bewijs laat zien van nieuwe ontstekingsgebieden.
- In de meeste gevallen kan MS alleen worden gediagnosticeerd nadat de ziekte al een tijd zijn beloop heeft gehad.
- De diagnose van MS kan nooit met volledige zekerheid worden gesteld.
- De diagnose MS kan worden gesteld enkel op basis van de aanwezigheid van antilichamen in het cerebrospinale vocht (liquor) die alleen bij MS voorkomen.

**17. Welke van de volgende uitspraken over exacerbaties is niet juist? ***

Kies a.u.b. een van de volgende mogelijkheden:

- Exacerbaties zijn nieuwe symptomen die zich ontwikkelen binnen een paar dagen of weken.
- Exacerbaties zijn oude symptomen die voor slechts een paar uur opnieuw op de voorgrond treden.
- Exacerbaties zijn op de voorgrond tredende oude symptomen of nieuwe symptomen die minimaal 24 uur aanhouden.
- Het kan soms moeilijk zijn om exacerbaties te onderscheiden van de dagelijkse schommelingen in de MS-symptomen.

**18. Welke van de volgende uitspraken over MS-therapieën is niet juist? ***

Kies a.u.b. een van de volgende mogelijkheden:

- MS-therapieën hebben tot doel de ziekte te genezen.
- MS-therapieën werken het best wanneer er sprake is van Relapsing Remitting MS.
- MS-therapieën vertragen de progressie van de ziekte.
- MS-therapieën kunnen de frequentie van exacerbaties verminderen.

**19. Welke van de volgende uitspraken over ziekteremmende medicatie is niet**

**juist? ***

Kies a.u.b. een van de volgende mogelijkheden:

- Mitoxantrone is goedgekeurd voor de behandeling van Relapsing Remitting MS.
- Sommige Interferonen zijn goedgekeurd voor de behandeling van Secundaire Progressieve MS.
- Tysabri® (Natalizumab) is goedgekeurd voor de behandeling van Relapsing Remitting MS.
- Gilenya® (Fingolimod) is goedgekeurd voor de behandeling van Progressieve MS.

**Bedankt voor uw deelname aan deze enquête!**

Bu anket sizin MS hastalığı üzerine olan bilginizi ölçmektedir. Özellikle, MS tedavisinde yer alan immunoterapötik etkili ilaç kullanmaları düşünülen hastaların risk bilgilendirilmesi amaçlı bir eğitim programıdır. Soruları yanıtlamada zorluk çekebilirsiniz. Ancak özelllikle belirtmekte yarar var ki, buradaki amaç sizin ne kadar “iyi” ya da “kötü” olduğunuzu test etmek değildir.

Hastalık hakkındaki bilginizin sağlık uzmanları tarafından ölçülme gereksiniminden kaynaklanmaktadır.

Lütfen her soruyu dikkatlice okuyunuz ve her soru için sadece bir cevabı işaretleyiniz.

Lütfen aşağıdaki soruları okuyun v e dikkatlice okuyunuz ve sizce doğru olan tek bir cevabı işaretleyiniz.

1. **Ataklarla ilgili hangisi doğrudur?**

- MS seyri boyunca sık ataklar gelecekteki hızlı kötüleşmenin habercisidir.
- Hastalığın ilk 2 yılındaki sık ataklar gelecekteki hızlı kötüleşmenin habercisidir.
- Hastalık seyri boyunca az atak iyileşmenin habercisidir.
- Hastalığın her hangi dönemindeki ağır ataklar gelecekteki hızlı kötüleşmenin habercisidir.

1. **Manyetik Rezonans Görüntüleme (MR) ile ilgili aşağıdaki ifadelerden hangisi doğrudur?**

- Hastanın engellilik durumu MRI ile değerlendirilebilir.
- MRI da kontrastlanma akut iltihaplanmanın bir işaretidir.
- Bütün MS hastaları yılda en az bir kez MRI çektirmelidir.
- MR’daki iltihaplanma odakları ( beyaz noktalar) sinirlerde ve sinir hücrelerinde tahribatı gösterir.

1. **MS hastalığının seyri ile ilgili aşağıdaki ifadelerden hangisi doğrudur?**

- MS tanısı konduğunda seyri de bellidir.
- Genelde ataklarla giden relapsing formdan ilerleyici kronik forma dönüş ancak hastalık geçmişi ile değerlendirilebilir.
- MS hastalarının çok azı başlangıçta ataklarla giden relapsing form MS’dir.
- MS hastalık seyri tedavi kararını belirlemez.

1. **MS hastalığının seyri ile ilgili aşağıdaki ifadelerden hangisi doğrudur?**

- 100 hastanın 30’da 20 yıldan sonrada MS iyi seyirli MS olarak kalacaktır.
- İyi seyirli MS yoktur.
- Tüm MS hastaları eninde sonunda dikkati çekecek şekilde kötüleşecektir.
- MS’ de kötüleşme hastalık tipine bağımlı değildir (ataklar ya da ilerleyici form).

1. **MS’in uzun seyri ile ilgili aşağıdaki ifadelerden hangisi doğrudur?**

Çalışmalar gösteriyor ki; hiç immunoterapötik ilaç almamış 15 yıl sonra…

- 100 hastanın 90’da yürüme çok kısıtlıdır.
- 100 hastanın 70’de yürüme çok kısıtlıdır.
- 100 hastanın 50’de yürüme çok kısıtlıdır.
- 100 hastanın 20’de yürüme çok kısıtlıdır.

1. **EDSS ölçeği ile ilgili aşağıdaki ifadelerden hangisi doğrudur?**

- EDSS ölçeği hastalık kötüleşmesini gösterebilir.
- EDSS 4 ile 7 arasındaki bir EDSS değeri başlıca hastanın yürüyebildiğini gösterir.
- EDSS değerlerindeki artış engellilikteki artışı yansıtır.
- Görme ve bilişsel yeteneklerdeki değişiklikler EDSS ile değerlendirilebilir.

1. **Aşağıdaki ifadelerden hangisi doğrudur?**

Rasgele yapılan plasebo kontrollü çift kör çalışma…

- önceki ilaç ile yeni ilacın karşılaştırıldığı bir çalışmadır.
- bir ilacın boş ilaç (plasebo) ile test edildiği bir çalışmadır. Hastalar çalışma gruplarına rasgele alınırlar. Ne hasta ne de doktor kimin hangi ilacı alacağını bilmezler.
- bir ilacın plasebo’ya karşı test edildiği bir çalışmadır. Hastalar çalışma gruplarına rasgele alınırlar. Çalışmadaki doktorlar hastalara hangi maddenin verileceği hakkında bilgilendirilir.
- hastaların gözlerinin iki kat bağlandığı ve farklı ilaçların denetildiği bir çalışmadır.

1. **Aşağıdaki 3 soru MS’de interferon çalışmalarındaki bozukluk stabilizasyonuyla ilgilidir (8a,8b,8c). Lütfen tüm 3 soruyu yanıtlayınız.**
2. **Plasebo teavilerinin sonuçları ile ilgili aşağıdaki ifadelerden hangisi doğrudur?**

Plasebo alırken MS stabilizasyonu:

Relapsing MSli 100 kişi düşünelim ve bunların hepsi yılda 2 kez atak geçirsin.. Placebo tedavisi ile geçen 2 yıldan sonra bu 100 kişiden kaçı hastalıkta hiçbir ilerleme gözlemez?

- yaklaşık 100 kişiden 15’i
- yaklaşık 100 kişiden 25’i
- yaklaşık 100 kişiden 40’i
- yaklaşık 100 kişiden 70’i
- yaklaşık 100 kişiden 80’i

1. **İnterferon çalışmalarının sonuçları ile ilgili aşağıdaki ifadelerden hangisi doğrudur?**

Interferon alırken MS stabilizasyonu:

Relapsing MSli 100 kişi düşünelim ve bunların hepsi yılda 2 kez atak geçirsin.. Interferon terapisi ile geçen 2 yıldan sonra bu 100 kişiden kaçı hastalık sabit kalsın?

about 15 of those 100

- yaklaşık 100 kişiden 15’i
- yaklaşık 100 kişiden 25’i
- yaklaşık 100 kişiden 40’i
- yaklaşık 100 kişiden 70’i
- yaklaşık 100 kişiden 80’i

1. **Interferon ve Placebo tedavilerini karşılaştıracak olursak aşağıdaki ifadelerden hangisi doğrudur?**

Farklı bir şekilde sorulursa: 8a sorusunun yanıtında 100 hastanın kaçında 2 yıl süreyle hastalık tedavisiz stabil kalacağını belirttiniz. Şimdi , buna ek olarak interferon terapisideı kaç hasta stabil kalır?

- yaklaşık 100 kişiden 10’i
- yaklaşık 100 kişiden 25’i
- yaklaşık 100 kişiden 45’i
- yaklaşık 100 kişiden 55’i
- yaklaşık 100 kişiden 65’i

1. **Farklı hastalık seyirleri için uygulanan terapilerle ilgili aşağıdaki ifadelerden hangisi doğrudur?**

- MS’in ilk belirtilerinin ortaya çıkmasına ilişkin tatmin edici terapi çalışmaları yoktur.
- MS’in nüksetmesine ilişkin tatmin edici terapi çalışmaları yoktur.
- Sekonder progresif MS’e ilişkin tatmin edici terapi çalışmaları yoktur.
- Primer progresif MS’e ilişkin tatmin edici terapi çalışmaları yoktur.

1. **Copaxon^®^ (Glatiramer acetate) tedavisi ile ilgili aşağıdaki ifadelerden hangisi doğrudur?**

- The Copaxon**^®^**’un atakları azaltıcı etkisi Interferon’unki ile hemen hemen aynıdır.
- The Copaxonu**^®^**n atakları azaltıcı etkisi Interferon’unkinden daha fazladır.
- Copaxonun**^®^** hastalığın nüksetmesinin azalması üzerineki etkisi Interferon’unkinden daha azdır.
- Copaxon**^®^** ve Interferon tedavilerinin hastalığın nüksetmesinin azalması üzerineki etkilerini karşılaştırmak imkansızdır.

1. **Interferon tedavisinin grip benzeri yan etkileri ile ilgili aşağıdaki ifadelerden hangisi doğrudur?**

- Interferon terapisi uygulanan bütün MS hastalarında terapi boyunca en az bir kere grip benzeri belirtiler ortaya çıkar.
- Interferon terapisinin başlangıcında grip benzeri belirtiler ortaya çıkar.
- Interferon terapisinin başlangıcında 100 hastadan 50’inde grip benzeri belirtiler ortaya çıkar.
- Interferon terapisinin başlangıcında 100 hastadan 10‘nda grip benzeri belirtiler ortaya çıkar.

1. **MS tabletleriyle ilgili aşağıdaki ifadelerden hangisi doğrudur?**

- Gilenya^®^ (Fingolimod) Tysabri®’den daha etkilidir.
- Gilenya^®^ (Fingolimod) az yan etkilere sahiptir.
- Tecfidera^®^ (Fumarat) etkinliği interferonlara benzer.
- Aubagio^®^ (Teriflunomide) interferonlardan daha etkilidir.

1. **Tysabri^®^ (Natalizumab) ile ilgili aşağıdaki ifadelerden hangisi doğrudur?**

- Tysabri® tedavisinin iki yılı boyunca MS’li 100 kişiden yaklaşık 40’unda terapinin sonucu olarak hastalık kötüleşmez.
- Tysabri® her 100 hastanın 50’inden fazlasında ani yan etkilere yol açar.
- Natalizumab ile tedavi edilen 1000 hastadan yaklaşık 3’ünde a bir viral beyin iltihabı (PML) görülür.
- Sekonder progresif MS’de Natalizumab ve Interferons seçime bağlı tedavilerdir. Non ritrovo questa quinta frase negli altri paesi
- İnterferonlara ek olarak, sekonder progresif MS’de Tysabri ilk seçenektir.

1. **Aşağıdakilerden hangisi MS ve gebelikle ilgili olarak doğrudur?**

- Ataklar gebelikte artar.
- MS tedavisi sırasında doğum kontrolü şarttır.
- MS’li ebeveyni olan 50 çocuktan biri bu hastalığa sahip olacaktır.
- Emzirme atak riskini arttırır.

1. **Tamamlayıcı tedaviler ve nutrisyonel desteklerle ilgili aşağıdakilerden hangisi doğrudur?**

- Çalışmalar enzim tedavilerinin atak oranını azalttığını göstermişlerdir.
- Çalışmalar doymamış yağların ( balık yağı,çuha çiçeği gibi) hastalığın ilerlemesini azalttığını göstermişlerdir.
- Çalışmalarda tamamlayıcı tedaviler ve nutrisyonel desteklerin hastalık aktivitesini etkilediğine dair kesin kanıtlar yoktur.
- Çalışmalar vitamin D'nin nüks oranlarını azalttığını göstermiştir.

**Şimdi, aşağıdaki ifadeler için lütfen yanlış olduğunu düşündüğünüz bir cevabı işaretleyin.**

1. **Hastalığın teşhisi ile ilgili aşağıdaki ifadelerden hangisi yanlıştır?**

- Tipik şikayetler ve eş zamanlı ya da daha sonra çekilen MRI da yeni iltihablı bir alan görülürse, MS teşhisi konulabilir.
- MS teşhisi sadece hastalığın seyrinden konulabilir.
- MS hastalığı kesin olarak teşhis edilemez.Bazen MS tanısı koymak çok zordur.
- MS teşhisi beyin-omurilik sıvısı içinde MS’e özgü antikorların varlığındakonulabilir.

1. **Nüksetmek ile ilgili aşağıdaki ifadelerden hangisi yanlıştır?**

- Ataklar genellikle günler veya haftalar içerisinde gelişen yeni bulgulardır.
- Ataklar sadece birkaç saat için olabilen daha önceki şikayetlerdir.
- Ataklar en az 24 saat süren eski bulguların kötüleşmesi ya da yeni ortaya çıkan şikayetlerdir.
- Bazen atakları MS bulgularının günlük dalgalanmalarından ayırt etmek zordur.

1. **MS tedavileri ile ilgili aşağıdaki ifadelerden hangisi yanlıştır?**

- MS tedavileri hastalığı tedavi etmeyi amaçlar.
- MS tedavileri daha çok relapsing MS’de etkilidir.
- MS tedavileri hastalığın ilerlemesini yavaşlatabilir.
- MS tedavileri hastalığın tekrarlama oranını azaltabilir.

1. **İmmunoterapi ile ilgili aşağıdaki ifadelerden hangisi yanlıştır?**

- Mitoxantrone relapsing MS tedavisi içn ruhsatlıdır.
- Bazı interferon ilaçları sekonder progresif MS tedavisi için onayıdır.
- Tysabri^®^ (Natalizumab) relapsing MS tedavisi için ruhsatlandırılmıştır.
- Gilenya^®^ (Fingolimod) kronik MS tedavisi için ruhsatlandırılmıştır.

**Yardımın için çok teşekkürler!**
