# Supplementary material for: Risk knowledge of people with relapsing-remitting multiple sclerosis – Results of an international survey
Source: PLoS One. 2018 Nov 29;13(11):e0208004. doi: 10.1371/journal.pone.0208004 (PMC6264873; doi:10.1371/journal.pone.0208004)
Supplement: S2 Appendix — (DOCX) [file pone.0208004.s006.docx]

# S2 Appendix. Electronic Control Preference Scale distribution by country.

# Table 1. Electronic Control Preference Scale distribution by country according to 3 categories of role responses.

|  | Total (N=364) | Germany (N=119) | Netherlands (N=130) | Italy (N=54) | Serbia (N=61) |
| --- | --- | --- | --- | --- | --- |
|  | N (%) |  |  |  |  |
| Active | 166 (46) | 77 (65) | 63 (48) | 9 (17) | 17 (28) |
| Collaborative | 155 (43) | 33 (28) | 57 (44) | 36 (67) | 29 (48) |
| Passive | 43 (12) | 9 (8) | 10 (8) | 9 (17) | 15 (25) |

# Table 2. Electronic Control Preference Scale distribution by country according to 6 categories of role responses.

|  | Total (N=364) | Germany (N=119) | Netherlands (N=130) | Italy (N=54) | Serbia (N=61) |
| --- | --- | --- | --- | --- | --- |
|  | N (%) |  |  |  |  |
| Active-active | 64 (18) | 30 (25) | 24 (18) | 4 (7) | 6 (10) |
| Active-collaborative | 102 (28) | 47 (39) | 39 (30) | 5 (9) | 11 (18) |
| Collaborative-active | 113 (31) | 26 (22) | 46 (35) | 26 (48) | 15 (25) |
| Collaborative-passive | 42 (12) | 7 (6) | 11 (8) | 10 (19) | 14 (23) |
| Passive-collaborative | 21 (6) | 7 (6) | 5 (4) | 7 (13) | 2 (3) |
| Passive-passive | 22 (6) | 2 (2) | 5 (4) | 2 (4) | 13 (21) |
