# Supplementary material for: Risk knowledge of people with relapsing-remitting multiple sclerosis – Results of an international survey
Source: PLoS One. 2018 Nov 29;13(11):e0208004. doi: 10.1371/journal.pone.0208004 (PMC6264873; doi:10.1371/journal.pone.0208004)
Supplement: S1 Table — (DOCX) [file pone.0208004.s002.docx]

# S1 Table. Questionnaires administered in each country.

| Instrument | Germany | Italy | The Netherlands | Serbia | Spain | Turkey |
| --- | --- | --- | --- | --- | --- | --- |
|  | N | | | | | |
| RIKNO 2.0 | 184 | 84 | 133 | 105 | 279 | 201 |
| eCPS* | 118 | 58 | 130 | 61 | - | - |
| MSKQ | 114 | 52 | 128 | - | - | - |

eCPS, electronic Control Preference Scale; MSKQ, Multiple Sclerosis Knowledge Questionnaire; RIKNO, Risk Knowledge Questionnaire in MS.

*Valid scores.
