# Supplementary material for: Risk knowledge of people with relapsing-remitting multiple sclerosis – Results of an international survey
Source: PLoS One. 2018 Nov 29;13(11):e0208004. doi: 10.1371/journal.pone.0208004 (PMC6264873; doi:10.1371/journal.pone.0208004)
Supplement: S2 Table — (DOCX) [file pone.0208004.s003.docx]

# S2 Table. Characteristics of participants by country.

|  |  | Total | Germany | Italy | Serbia | Spain | Netherlands | Turkey | *P* |
| --- | --- | --- | --- | --- | --- | --- | --- | --- | --- |
| Participants^a^ |  | 986 | 184 (19) | 84 (9) | 105 (11) | 279 (28) | 133 (14) | 201 (20) |  |
| Recruitment strategies |  |  | Homepage | Mailing lists, homepage | Personal contact, mailing list | Homepage | Mailing lists | Personal contact |  |
| Mother tongue^a^ |  | 940 (95) | 176 (96) | 83 (99) | 105 (100) | 249 (89) | 127 (96) | 200 (100) | <0.001 |
| Women^a^ |  | 755 (77) | 142 (77) | 78 (93) | 77 (74) | 213 (76) | 123 (93) | 122 (61) | <0.01 |
| Age (years)^b^ |  | 38.6 (18-67) | 38.2 (19-60) | 40.6 (26-64) | 37.0 (20-57) | 40.8 (18-67) | 40.6 (23-60) | 34.6 (18-62) | <0.01 |
| Disease duration (years)^b^ |  | 7.8 (0-37) | 5.5 (0-28) | 7.7 (1-30) | 8.6 (0-35) | 9.9 (0-37) | 4.7 (0-22) | 8.5 (0-32) | <0.01 |
| RRMS course^a,c^ |  | 934 (95) | 163 (89) | 79 (94) | 101 (96) | 265 (95) | 129 (97) | 197 (98) | <0.01 |
| Within a DMD decision making process^a^ |  |  |  |  |  |  |  |  |  |
|  | Start | 228 (23) | 55 (30) | 10 (12) | 42 (40) | 27 (10) | 28 (21) | 66 (33) | <0.001^d^ |
|  | Change | 430 (44) | 102 (55) | 38 (45) | 57 (54) | 101 (36) | 32 (24) | 100 (50) |  |
|  | Stop | 106 (11) | 27 (15) | 8 (10) | 6 (6) | 20 (7) | 10 (8) | 35 (17) |  |
|  | No decision making process | 222 (23) | -^e^ | 28 (33) | -^e^ | 131 (47) | 63 (47) | -^e^ |  |
| Previous DMD^a^ |  |  |  |  |  |  |  |  |  |
|  | Yes | 401 (41) | 90 (49) | 31 (37) | 18 (17) | 74 (27) | 72 (100) | 116 (58) | <0.001 |
|  | Missing values | 114 (11) | 0 | 1 (1) | 3 (3) | 48 (17) | 61 (46) | 1 (1) |  |
| Current DMD^a^ |  |  |  |  |  |  |  |  |  |
|  | Yes | 645 (65) | 123 (67) | 68 (81) | 56 (53) | 229 (82) | 40 (30) | 129 (64) | <0.001 |
|  | Missing values | 291 (30) | 55 (30) | 10 (12) | 42 (40) | 27 (10) | 91 (68) | 66 (33) |  |
| Current DMD since (months)^b^ |  | 44 (0-302) | 27 (0-168) | 42 (2-168) | 57 (2-130) | 50 (0-302) | 30 (0-84) | 49 (2-204) | <0.001 |
| Consulting physicians for MS^a^ |  |  |  |  |  |  |  |  |  |
|  | MS center | 594 (60) | 69 (38) | 72 (86) | 96 (91) | 115 (41) | 42 (32) | 200 (100) | <0.001 |
|  | Neurologist | 489 (50) | 140 (76) | 32 (38) | 12 (11) | 183 (66) | 115 (86) | 7 (4) | <0.001 |
|  | General practitioner | 82 (8) | 47 (26) | 5 (6) | 1 (1) | 10 (4) | 19 (14) | 0 | <0.001 |
| Perception of the severity of the disease^b,f^ |  | 37 (1-51) | 39 (1-51) | 38 (4-51) | 37 (3-51) | 36 (2-51) | 39 (1-51) | 36 (1-51) | <0.001 |
| Fear for wheelchair dependency^b,g^ |  | 42 (1-51) | 38 (1-51) | 44 (3-51) | 47 (6-51) | 42 (5-51) | 38 (5-51) | 43 (1-51) | <0.001 |
| PDDS^b^ |  | 2.54 (1-8) | 2.76 (1-8) | 2.13 (1-8) | 2.25 (1-7) | 2.58 (1-8) | 2.48 (1-7) | 2.64 (1-8) | 0.024 |
| Education^a^ |  |  |  |  |  |  |  |  |  |
|  | Graduate | 492 (50) | 90 (49) | 37 (44) | 60 (57) | 149 (53) | 56 (42) | 100 (50) | 0.15 |
| Living with:^a^ |  |  |  |  |  |  |  |  |  |
|  | Alone | 163 (17) | 44 (24) | 14 (17) | 17 (17) | 34 (12) | 21 (16) | 33 (16) | 0.049 |
|  | Partner | 628 (64) | 119 (65) | 54 (64) | 57 (54) | 173 (62) | 92 (71) | 131 (65) | 0.19 |
|  | Children | 322 (33) | 46 (25) | 20 (24) | 48 (46) | 82 (29) | 64 (48) | 63 (31) | <0.001 |
|  | Parents | 117 (12) | 7 (4) | 13 (16) | 27 (26) | 39 (14) | 1 (1) | 30 (15) | <0.001 |
|  | Other | 27 (3) | 5 (3) | 3 (4) | 2 (2) | 16 (6) | 0 (0) | 1 (1) | 0.004 |
| Risk attitude^h^ |  | 6.16 (1-11) | 5.27 (1-10) | 5.20 (1-11) | 6.02 (1-11) | 7.21 (1-11) | 6.89 (1-10) | 5.53 (1-11) | <0.001 |
| Correct medical data interpretion^a^ |  | 785 (80) | 155 (84) | 57 (68) | 98 (93) | 190 (68) | 116 (87) | 169 (84) | <0.001 |

P-values show the significances for the comparison between countries within the RIKNO 2.0 completers. DMD, disease modifying drug; MS, multiple sclerosis; PDDS, Patient Determined Disease Steps.

^a^ N (%)

^b^ Mean (range)

^c^ Relapsing Remitting Multiple Sclerosis, all other participants were newly diagnosed; having another disease course was an exclusion criterion

^d^ Comparison: decision making process: yes/ no only for Italy, Spain, and the Netherlands

^e^ Different inclusion criteria per country

^f^  1: MS is rather a benign disease; 51: a serious disease

^g^ 1: No problem at all; 51: worst thing imaginable

^h^ 1: Not at all open to risks; 10: very open to risks
