# Supplementary material for: Risk knowledge of people with relapsing-remitting multiple sclerosis – Results of an international survey
Source: PLoS One. 2018 Nov 29;13(11):e0208004. doi: 10.1371/journal.pone.0208004 (PMC6264873; doi:10.1371/journal.pone.0208004)
Supplement: S3 Table — (DOCX) [file pone.0208004.s005.docx]

**S3 Table. Item characteristics of the RIKNO 2.0 and MSKQ.**

| Instrument | Country | N | Mean item difficulty (SD, range) | Mean r_it(i)_ (range) | Cronbach alpha |
| --- | --- | --- | --- | --- | --- |
| RIKNO 2.0 | Total | 986 | 0.42 (0.46, 0.11-0.74) | 0.23 (0.00-0.38) | 0.66 |
|  | Germany | 184 | 0.53 (0.45, 0.12-0.89) | 0.21 (0.01-0.38) | 0.62 |
|  | Italy | 84 | 0.38 (0.43, 0.04-0.89) | 0.10 (-0.31-0.31) | 0.41 |
|  | Serbia | 105 | 0.56 (0.43, 0.04-0.96) | 0.27 (-0.35-0.49) | 0.72 |
|  | Spain | 279 | 0.36 (0.44, 0.08-0.81) | 0.18 (-0.07-0.33) | 0.57 |
|  | Netherlands | 133 | 0.44 (0.44, 0.05-0.83) | 0.09 (-0.15-0.27) | 0.36 |
|  | Turkey | 201 | 0.32 (0.42, 0.03-0.68) | 0.11 (-0.18-0.38) | 0.46 |
| MSKQ | Total | 294 | 0.78 (0.34, 0.27-100) | 0.21 (0.01-0.30) | 0.63 |
|  | Germany | 114 | 0.80 (0.33, 0.33-100) | 0.18 (0.00-0.35) | 0.58 |
|  | Italy | 52 | 0.68 (0.33,0.29-100) | 0.18 (-0.13-0.46) | 0.56 |
|  | Netherlands | 128 | 0.77 (0.34, 0.19-100) | 0.25 (-0.03-0.43) | 0.69 |

MSKQ, Multiple Sclerosis Knowledge Questionnaire; RIKNO, Risk knowledge in multiple sclerosis; SD, standard deviation.

Item characteristics of RIKNO 2.0 and MSKQ were calculated, including item difficulty (norms: 0.2-0.8), standard deviation (norms: >0.2), corrected item total correlations (r_it(i)_ norms: 0.3-0.5), Cronbach’s alpha (norms: >0.7, expected lower values due to multidimensional structure of the questionnaire), and normal distribution criteria (Kolmogorov-Smirnov-test, α=0.05, two-tailed).

All item difficulties and SDs of the RIKNO 2.0 and the MSKQ met expected criteria for the whole sample and per country. Item total correlations and Cronbach’s alpha were slightly below the expected range. Missing values for RIKNO 2.0 ranged between 0-8%. Mean of “don’t know” answers in MSKQ were 6% per country. The normal distribution criterion was not met in any country.
